# Development and validation of an explainable machine learning model to predict Delphian lymph node metastasis in papillary thyroid cancer: a large cohort study

**DOI:** 10.7150/jca.110141

**Published:** 2025-03-03

**Authors:** Jie Cui, Genglong Liu, Kai Yue, Yansheng Wu, Yuansheng Duan, Minghui Wei, Xudong Wang

**Affiliations:** 1 Department of Maxillofacial and Otorhinolaryngological Oncology, Tianjin Medical University Cancer Institute and Hospital, Key Laboratory of Basic and Translational Medicine on Head & Neck Cancer, Tianjin, Key Laboratory of Cancer Prevention and Therapy, Tianjin Cancer Institute, National Clinical Research Center of Cancer, Tianjin, 300060, PR China.; 2 Department of Head and Neck Surgery, National Cancer Center/National Clinical Research Center for Cancer/Cancer Hospital & Shenzhen Hospital, Chinese Academy of Medical Sciences and Peking Union Medical College, Shenzhen, 518116, Guangdong Province, PR China.; 3 School of Medicine, Southern Medical University, Foshan, 528305, Guangdong Province, PR China.; 4 Editor Office, iMeta, Shenzhen, 518000, Guangdong Province, PR China.

**Keywords:** delphian lymph node metastasis, papillary thyroid cancer, machine learning approaches, prediction model, model interpretability

## Abstract

**Background:** The occurrence of papillary thyroid cancer (PTC) has risen substantially and tends to exhibit early-stage lymph node metastasis (LNM), increasing the risk of postoperative recurrence and decreasing survival. There is a lack of a machine learning (ML) model to predict delphian LNM (DLNM) in PTC. This investigation seeks to comprehensively assess the significance of standard clinical indicators for DLNM prediction, while constructing a dependable and widely applicable ensemble ML framework to support surgical planning and therapeutic decision-making.

**Methods:** This investigation incorporated 1993 sequential PTC patients who underwent curative surgical procedures from 2020 to 2023. Based on the time to surgery, we divided the cohort into the training cohort (n=1395) and the validation cohort (n=598). The Boruta algorithm was applied to select feature variables, succeeded by the development of an innovative ML structure combining 12 ML techniques across 113 permutations to create a unified prediction model (DLNM index). ROC analysis, calibration curve, Bootstrapping, 10-fold cross validation, restricted cubic spline (RCS) regression, multivariable logistic regression, and subgroup analysis were utilised to evaluate the predictive accuracy and discriminative ability of the DLNM index. Model interpretation and feature impact visualisation were accomplished through the Shapley Additive Explanations (SHAP) methodology.

**Results:** Based on 14 features via the Boruta algorithm selection, we integrated them into 12 ML approaches, yielding 113 permutations, from which we identified the superior algorithm to establish a consensus ML-derived diagnostic model (DLNM index). The DLNM index exhibited excellent diagnostic values with a mean AUC of 0.763 in two cohorts and discriminative ability, serving as an independent risk factor (*P* < 0.001). It performed better in predicting performance and yielded a larger net benefit than the published model (*P* < 0.05). Bootstrapping and 10-fold cross validation, and subgroup analysis showed that the DLNM index was generally robust and generalisable. SHAP explains the importance of ranking features (tumour size, right 4 region LN, FT4, TG, and T3) and visualises global and individual risk prediction. RCS regression suggested a nonlinear link between the DLNM index, TG, tumour size, FT3, and DLNM risk.

**Conclusion:** An optimised explainable model (DLNM index) comprising 12 clinical features based on multiple ML algorithms was constructed and validated to provide an economical, readily available, and precise diagnostic instrument for DLNM in PTC, which has potential implications for clinical practice. The SHAP explanation and RCS regression quantify and visualise tumour size and FT4 as the most important variables that increase DLNM risk.

## Introduction

The incidence of thyroid cancer has substantially risen over recent decades, with papillary thyroid cancer (PTC) comprising the majority at roughly 80%-90% 1,2. While PTC patients experience low mortality rates, metastatic spread to cervical lymph nodes (LN) occurs promptly and commonly, with lymph node metastasis (LNM) rates ranging from 20% to 90% 3. The central cervical nodes, encompassing right and left paratracheal, pretracheal, and prelaryngeal LN, represent the primary sites of nodal metastasis in PTC cases 4. Current research indicates that cervical LNM functions as a negative predictor associated with locoregional recurrence and distant metastasis 5,6. Therefore, precise preoperative assessment of LNM extent and distribution is essential for developing targeted prevention and intervention approaches.

The Delphian LN (DNL), also known as the prelaryngeal LN, is a central regional LN component, typically manifesting as an individual node or nodal cluster, facilitating lymphatic flow from the throat and thyroid structures 7. Contemporary research suggests that DLN metastasis (DLNM) serves as a reliable indicator of regional nodal involvement and reoccurrence in diverse malignant head and neck tumours encompassing PTC 8,9. Although DLNM is one of the common local LNMs in PTC, it has often been overlooked in the past, and its significance in PTC has recently garnered increased recognition. Investigations demonstrate that DLNM exhibits aggressive characteristics and suggests elevated recurrence probability in PTC 8,10. Given that the location of the DLN is hidden, it is often difficult to identify metastasis based on imaging. Additionally, there remains debate regarding whether prophylactic central neck LN dissection should be performed in clinically LN-negative PTC cases, while current guidelines advise against prophylactic DLN dissection for PTC patients. More importantly, with the development of endoscopic thyroidectomy, there has been an increase in the development of endoscopic procedures such as transoral vestibular approach, trans-chin approach, trans-axillary approach, trans-subclavian approach, and trans-thoracic breast approach; however, there are difficulties in the presence of DLN clearance in these endoscopic thyroidectomy procedures, and surgeons tend to overlook the DLN clearance. Consequently, developing non-invasive, reliable, and accessible evaluation methods for preoperative DLNM identification becomes crucial for surgical planning.

Prediction models could potentially aid in important decisions, such as the identification of PTC patients who may have a high DLNM risk based on clinical features. Only two risk prediction models 11,12 have been developed to assess DLNM in PTC patients. Nevertheless, these models' transferability and robustness require additional verification, given that the sample sizes of these studies were small, the overall methodological quality (methods of model construction, overfitting problems, model evaluation, model comparison and model interpretation) was poor and lack of important biochemical markers. Most importantly, current models are unsuitable for identifying high-risk DLNM and providing preoperative decision support for prophylactic DLN dissection, as all models were developed in PTC patients, including the postoperative variable. Contemporary advancements in computational capabilities and large-scale data processing have facilitated machine learning (ML) applications that increasingly surpass conventional approaches in addressing complex decision-making challenges 13, which is especially important in this age of precision medicine.

In this large retrospective cohort study, we formulated an innovative multi-criteria decision-making system through the leave-one-out cross-validation (LOOCV) framework for diagnostic prediction by converting 12 classical ML algorithms into 113 combinations. Analogous to clinical practice's multidisciplinary treatment approach, which synthesises various expert perspectives to achieve a unified medical determination, this framework strives to enhance diagnostic accuracy and optimise DLNM prediction performance. Generally speaking, we systematically evaluate the ability of preoperative routine clinical features to predict DLNM of PTC and develop a robust and generalisable DLNM model. Then, we perform various comprehensive statistical methods for model evaluation, model comparison and model interpretation.

## Materials and Methods

The Ethics Review Committee of the National Cancer Center/National Clinical Research Center for Cancer/Cancer Hospital & Shenzhen Hospital, Chinese Academy of Medical Sciences and Peking Union Medical College authorised this retrospective analysis (Approval No. KYKT2022-7-1), adhering to the World Medical Association Declaration of Helsinki's ethical principles. The institutional review board waived the requirement for informed consent, and data were identified. This research follows the guidelines outlined in the Transparent Reporting of a Multivariable Prediction Model for Individual Prognosis or Diagnosis (TRIPOD).

### Study design

The research design for this study comprises 5 steps: feature selection, model construction and validation, model evaluation, model comparison, and model interpretation. Initially, the Boruta algorithm was applied to select feature variables, followed by developing and validating an innovative LOOCV framework integrating 12 ML techniques, resulting in 113 combinations to construct a consensus prediction model. Furthermore, the performance, discrimination and stability of the model were comprehensively evaluated. Meanwhile, we compared it with the published model. Lastly, we employed the Shapley Additive Explanations (SHAP) algorithm and restricted cubic spline (RCS) regression to illuminate and demonstrate the predictive variables' importance and identify nonlinear associations among risk indicators.

### Setting and population

We executed a retrospective cohort analysis of consecutive individuals with PTC who received curative surgical treatment between January 2020 and December 2023. The inclusion criteria were as follows: (a) adult patients (> 18 years); (b) individuals with PTC diagnosed by multidisciplinary teams, encompassing clinicians, radiologists and pathologists; (c) surgical procedures following Chinese Thyroid Association guidelines 14,15, lobectomy or total thyroidectomy combined with central neck dissection (CND) was executed, and lateral neck dissection (LND) was performed if necessary. The exclusion criteria were as follows: (a) a previous history of thyroidectomy; (b) history or coexistence of other head and neck cancers; (c) DLN was not detected by pathological examination; and (d) incomplete or indeterminate clinicopathologic information. Following these criteria, 1993 PTC patients qualified for the investigation. The participants were chronologically allocated into a training cohort (January 2022 to December 2023; n = 1395) and a validation cohort (January 2020 to December 2021; n = 598) according to the time to surgery. The trial was registered with ClinicalTrials.gov (NCT03604601).

Detailed surgical procedure, data extraction and outcomes are available in the **Supplementary appendix**.

### Statistical analysis

**Description and comparison of clinicopathologic features:** Baseline data analysis of patients commenced with normality assessment of quantitative measurements. Skewed data were described using the median (interquartile range [IQR]), and group comparisons were executed via the Mann-Whitney U tests. Normally distributed continuous data were denoted as Mean±standard deviation (SD), and comparisons between groups were executed utilising independent samples t-tests. Categorical variables were denoted as frequency (percentage, %), with comparisons executed via the chi-square test or Fisher's exact test when applicable.

**Feature selection:** The meticulous identification of appropriate variables is essential in ensuring diagnostic model effectiveness. Consequently, we implemented the Boruta algorithm, which utilises random forest classification to conduct variable selection across multiple high-dimensional, multivariate datasets 17. This methodology enables the identification of the most significant variables that enhance both model precision and reliability.

**Model construction and validation:** Based on characterisation variables through Boruta algorithm selection, we developed and validated a consensus diagnostic model for DLNM. The following procedure was performed : (1) Integration of 12 traditional algorithms: Stepglm (glmnet, version 4.1.8), gradient boosting machine (GBM) (gbm version 2.1.8.1), Linear Discriminant Analysis (LDA) (MASS version 7.3.60), eXtreme Gradient Boosting (XGBoost) (xgboost version 1.7.5.1), NaiveBayes (BART version 1.7.5.1), SVM (e1071 version 1.7.13), random forest (RF) (randomForestSRC version 3.2.2), glmBoost (mboost, version 2.9.8), LASSO (glmnet, version 4.1.8), Partial Least Squares Regression for Generalized Linear Models (plsRglm) (plsRglm version 1.5.1), ridge regression (glmnet, version 4.1.8), elastic network (Enet) (glmnet, version 4.1.8). Among these, LASSO, RF, Stepglm, and glmBoost possess feature selection attributes. Furthermore, 113 algorithmic combinations were established as predictive frameworks utilising the LOOCV structure. (2) Subsequently, for the training cohort, we employed 113 combinations of ML to generate classifiers independently using selection features. (3) Lastly, we computed the DLNM index for each group in the validation cohort using the model obtained from the training cohort. The optimal consensus diagnostic model for DLNM was determined by evaluating the average area under the curve (AUC) of both cohorts while considering model parsimony and generalizability.

**Model evaluation:** The discriminatory ability of the DLNM index was examined utilising Receiver operating characteristic (ROC) curves analysis alongside AUC measurements. In addressing model performance assessment, the data imbalance issue was tackled through adaptive synthetic sampling, employing a 0.5 balancing ratio. The predictive capabilities were measured using multiple evaluation metrics, encompassing accuracy, prevalence, recall, F1-score, Matthews correlation coefficient (MCC), precision, specificity, false negative rate (FNR), and false positive rate (FPR). To examine the DLNM index calibration, plots were generated showing the relationship between predicted probabilities and actual observed probabilities. The clinical value of the DLNM index was investigated through decision curve analysis (DCA), which served as a comprehensive approach to assess and contrast the DLNM index against the baseline by calculating net benefits across various threshold probabilities.

Additionally, to investigate the degree of multicollinearity among variables in the DLNM index, the variance inflation factor (VIF) was computed in the multiple linear regression analysis. If VIF was > 3 or tolerance < 0.1, then multicollinearity was high.

Finally, in order to evaluate and improve the reliability and generalisation ability of the DLNM index, Bootstrap and 10-fold cross-validation were used 18. Cross-validation represents an analytical approach that evaluates and compares the performance of different models on a finite data set by splitting the data set into training and testing components to verify model outcomes. By using cross-validation, researchers can avoid relying on a single experiment and can therefore better evaluate the generalisation ability of the model.

**Multivariable logistic regression (MLR), subgroup analysis and interaction effect:** MLR was utilised to ascertain the DLNM index as an independent risk factor for DLNM in PTC patients. To investigate possible differences among distinct subpopulations, subgroup analysis was executed by stratifying patients according to sex, age, time to diagnose the tumour, tumour size, PTC subtype, fibrosis, small foci of squamous lesions, hashimoto thyroiditis, pT stage, pN stage, pM, pTNM, multifocal, vascular invasion, intra-glandular dissemination, capsular invasion, extracapsular spread, trachea invasion, nerve invasion, superior mediastinal metastasis, left CLN metastasis, left 3 region LN metastasis, left 4 region LN metastasis, right CLN metastasis, right 2 region LN metastasis, right 3 region LN metastasis, right 4 region LN metastasis. The link between the DLNM metric and these stratification parameters in subgroup assessment was examined utilising likelihood ratio testing.

**Model comparison:** Multiple ROC analysis was executed to evaluate and contrast the discriminative capabilities of the DLNM index with other published models 11,12. DCA was implemented to assess the net benefit of the DLNM index relative to existing published models.

**Model interpretability:** To help interpret the model, the SHAP method was used 19. The SHAP value 20 was computed to evaluate the significance of individual clinical characteristics and deliver a numerical analysis of the relationship connecting DLNM with all 12 attributes based on the pre-model. Furthermore, the SHAP method supplied both comprehensive and specific interpretations for model clarification. The comprehensive interpretation could present reliable and precise attribution measurements for each characteristic within a model to illustrate the connections between input attributes and DLNM. The specific interpretation could reveal a particular forecast for an individual PTC through data input. SHAP dependence visualisations can reveal meaningful nonlinear correlations between predictive variables and DLNM.

**Associations between DLNM index as well as predictive variables and DLNM:** To further explore the importance of characteristics in the predictive model, we analysed both linear and nonlinear associations between the DLNM index as well as predictive variables and DLNM. Linear associations were assessed using univariable logistic regression models, where each model included DLNM and clinical variables as predictors. Linearity was inspected by applying the Wald test to the regression coefficient. Additionally, we explored the potential nonlinear associations using RCS regression between the DLNM index as well as predictive variables and DLNM.

Statistical computations were performed utilising SPSS statistics 22.0, DecisionLinnc1.0 software (Python) 21, and R software (R version 4.3.1). DecisionLinnc1.0 functions as an integrated platform that combines various programming language environments, facilitating data processing, analysis, and ML through a graphical user interface. Statistical significance was established at a two-sided *P* < 0.05.

## Results

### Study population and patient characteristics

There were a sum of 1993 patients in the study cohort. DLNM was observed in 405 (405/1993=20.32%) patients. This investigation carried out an initial comparison among two cohorts: the training cohort (n=1395) and the validation cohort (n=598). The comparison focused on their preoperative 28 baseline characteristics and DLNM rate (288/1395=20.65% *vs* 117/598=19.57%; *P=*0.583) (**Supplementary Material 1**). No variations in preoperative clinical characteristics were detected between the training cohort and validation cohort except for age (*P* < 0.001) and T4 (*P =* 0.013).

Sequentially, we compared preoperative 28 baseline characteristics between the DLNM cohort and the non-DLNM cohort in the entire cohort, training cohort, and validation cohort (**Table 1**). It was observed that the B ultrasound tumour size, left CLN suspicious metastasis, left 3 regions LN suspicious metastasis, left 4 region LN suspicious metastasis, right CLN suspicious metastasis, right 2 region LN suspicious metastasis, right 3 region LN suspicious metastasis, right 4 region LN suspicious metastasis, CT CLN suspicious metastasis, lateral cervical LN suspicious metastasis, TG, T3, FT3, and FT4 were elevated in the DLNM cohort (all *P* < 0.05). In contrast, the time to diagnose the tumour was shortened in the DLNM cohort versus the non-DLNM cohort (*P* < 0.05). These findings suggest a notable link between these routine preoperative parameters and DLNM occurrence in PTC.

### Variable selection

**Figure 1:**
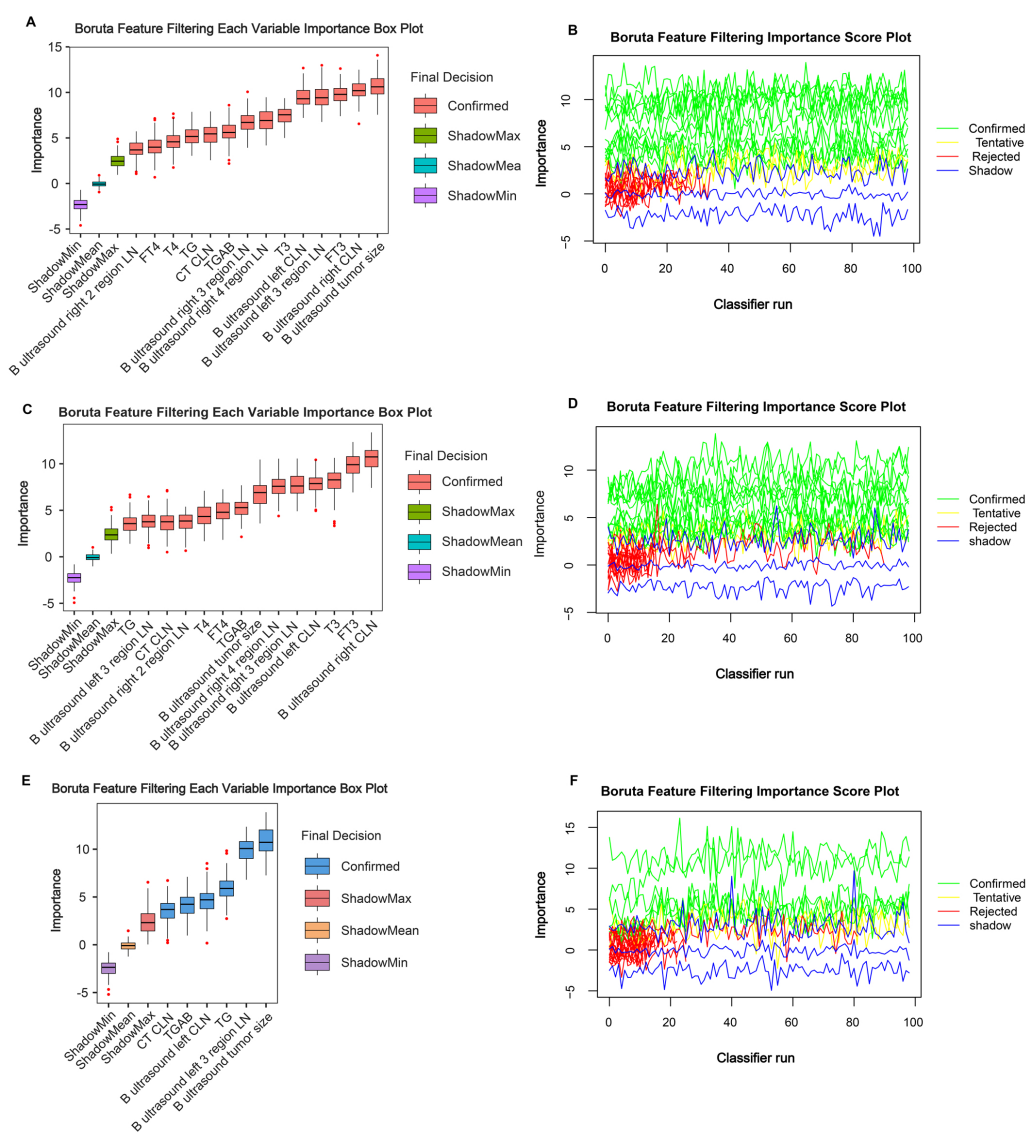
Important characteristic variables identified by the Boruta algorithm. The horizontal axis shows the names of variables, and the vertical axis shows the Z-score of variables. The boxplot shows the Z-score of variables during the model calculation process. (A, B) Entire cohort. (C, D) Training cohort. (E, F) Validation cohort.

To control for confounding factors and find robust variables, the Boruta algorithm facilitated a comprehensive evaluation of the 28 independent variables. Through the Boruta algorithm's selection process, we confirmed 14 variables in the entire cohort (**Figure 1A and 1B**), 14 variables in the training cohort (**Figure 1C and 1D**), and 6 variables in the validation cohort (**Figure 1E and 1F**). We take the intersection of three results to acquire 14 indicators (B ultrasound tumour size, left CLN metastasis, left 3 region LN metastasis, left 4 region LN metastasis, right CLN metastasis, right 2 regions LN metastasis, right 3 regions LN metastasis, right 4 regions LN metastasis, CT CLN metastasis, TG, TGAB, T3, T4, FT3, and FT4) shared by ≥ two results. Detailed variable selection information is presented in **Supplementary Material 2.** Noticeably, tumour size and TG features exhibited a higher predictive capacity for DLNM.

### Optimised model construction and validation

**Figure 2:**
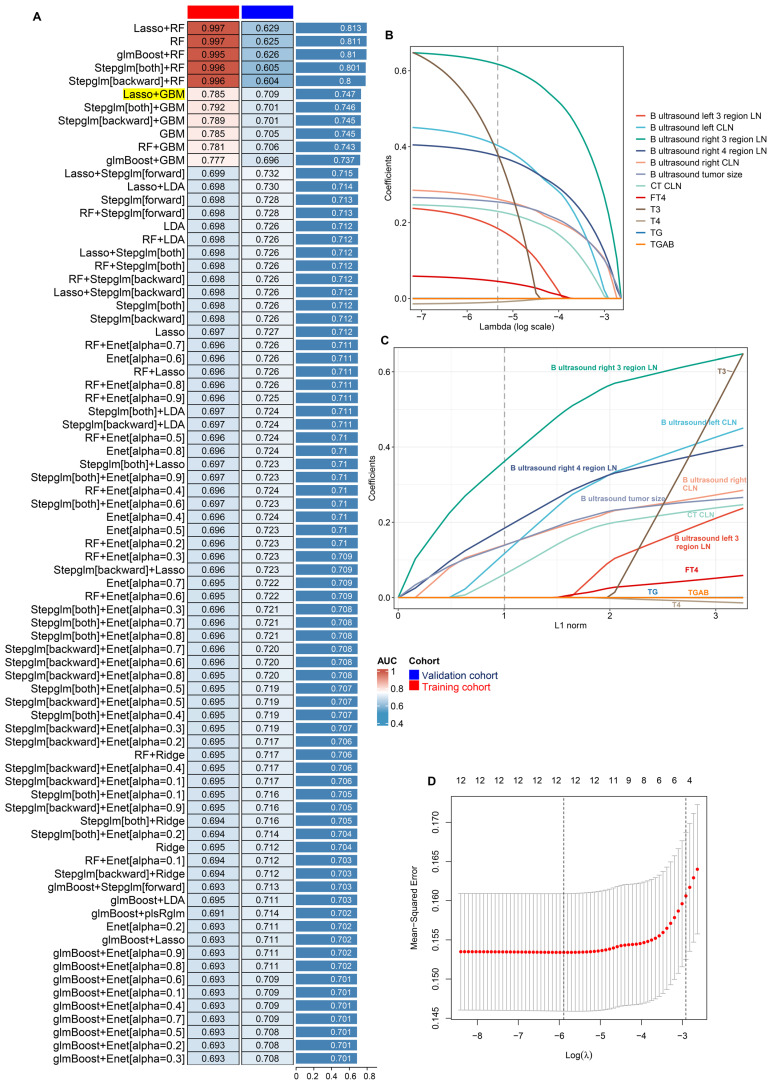
Establishment and validations of a consensus diagnostic model for DLNM via 12 the machine learning (ML)-based integrative procedure. (A) A total of 113 ML algorithm combinations of prediction models using the LOOCV framework and further calculated the area under curve (AUC) of each model in all datasets in Figure S2. (B, C) Lasso coefficient profiles of the 12 predictors. A vertical line is drawn at the optimal value by 1 - s.e. criteria and results in 12 non-zero coefficients (B ultrasound tumor size, left CLN metastasis, left 3 region LN metastasis, right CLN metastasis, right 3 region LN metastasis, right 4 region LN metastasis, CT CLN metastasis, TG, TGAB, T3, T4, and FT4). (D) Lasso was used to identify candidate features with 10-fold cross-validation. The Y-axis shows mean-square error and the X-axis is Log (λ), dotted vertical lines represent minimum and 1 standard error values of λ. The features selected at minimum standard error values of λ were finally used for DLNM model.

To establish a reliable diagnostic model for DLNM, we integrated 14 feature variables into our analytical system (LOOCV framework). We constructed predictive models utilising 12 ML algorithms and 113 algorithmic combinations, implementing 10-fold cross-validation within the training cohort. For training and validation cohorts, the AUC value was computed for the individual algorithms, as well as the average AUC in the two cohorts. We note that the “Lasso+RF”, “RF”, “glmBoost + RF”, “Stepglm[both]+RF”, and “Stepglm[backward] + RF” models have very high AUC values in the training cohort, but their performances degrade in the validation cohort. This suggests an overfitting phenomenon. Thus, the most effective model emerged as an integration of Lasso and GBM with a higher average AUC (0.763), least variable and more stability from the two cohorts, as illustrated in **Figure 2A and Figure S2**. In the Lasso regression, the optimal λ (λ =12; B ultrasound tumour size, left CLN metastasis, left 3 region LN metastasis, right CLN metastasis, right 3 region LN metastasis, right 4 region LN metastasis, CT CLN metastasis, TG, TGAB, T3, T4, and FT4) was obtained when the mean-squared error reached its minimum within the LOOCV framework (**Figure 2B-D**). Eventually, we developed a unified diagnostic model designated as the DLNM index, incorporating these 12 features. Comprehensive information regarding feature selection for each model, predictive classification, and individual patient risk scores is available in **Supplementary material 3**.

### Model evaluation and cross-validation

First, the examination of multi-collinearity among the 12 variables showed a VIF score of less than 2 and tolerance exceeding 0.5, suggesting no substantial multi-collinearity issues between the variables in the DLNM model (**Supplementary material 4**).

**Figure 3:**
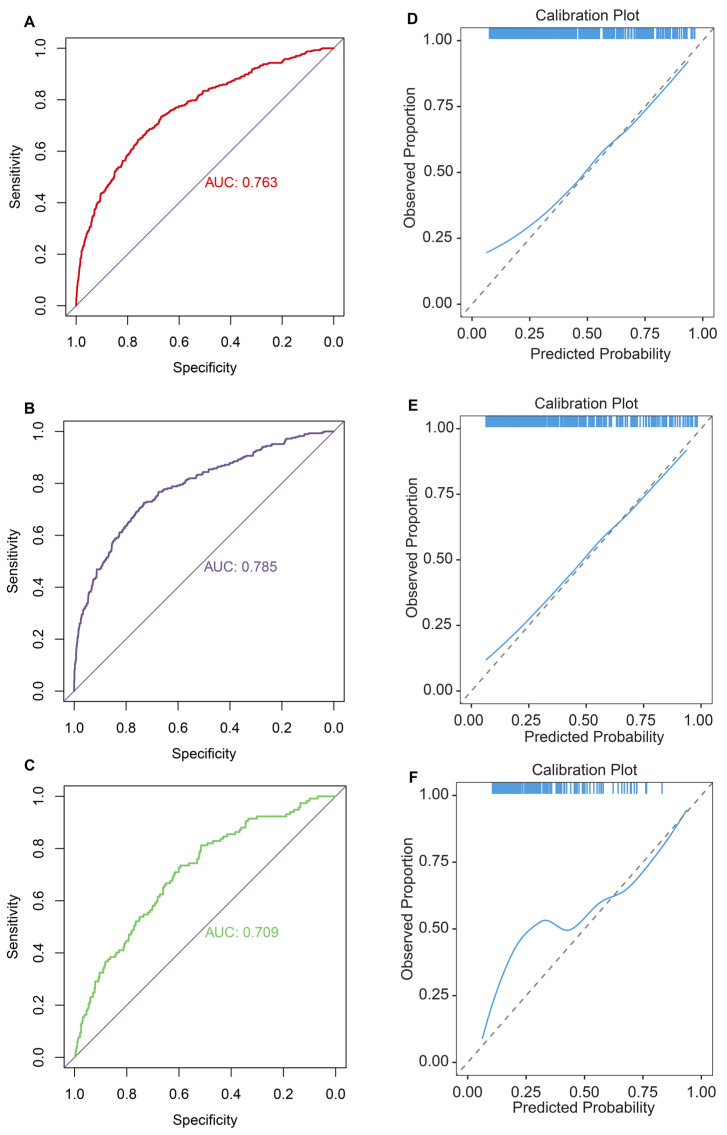
Evaluation of diagnostic value and fitting ability of DLNM index. (A-C) Receiver operating characteristic (ROC) curves with AUC values to evaluate predictive efficacy of DLNM index in entire cohort (A), training cohort (B), validation cohort (C). (D-F) Calibration curves for DLNM index in entire cohort (D), training cohort (E), validation cohort (F). X-axis is predicted probability of DLNM. Y-axis is observed probability of DLNM.

Second, ROC curves suggested that the DLNM index exhibited exceptional predictive capability, achieving an AUC of 0.763 in the entire cohort (**Figure 3A**), an AUC of 0.785 in the training cohort (**Figure 3B**), and an AUC of 0.709 in the validation cohort (**Figure 3C**). The data imbalance was corrected after the adaptive synthetic sampling method; the accuracy, prevalence, recall, F1-score, MCC, precision, specificity, FNR, and FPR in three cohorts were calculated and presented in **Table 2**. All three cohorts showed high predictive performance. The calibration curves aligned closely with the reference line (y=x) in three cohorts (**Figure 3D-F**), indicating that the predicted probability of the model fitted well with the actual probability.

**Figure 4:**
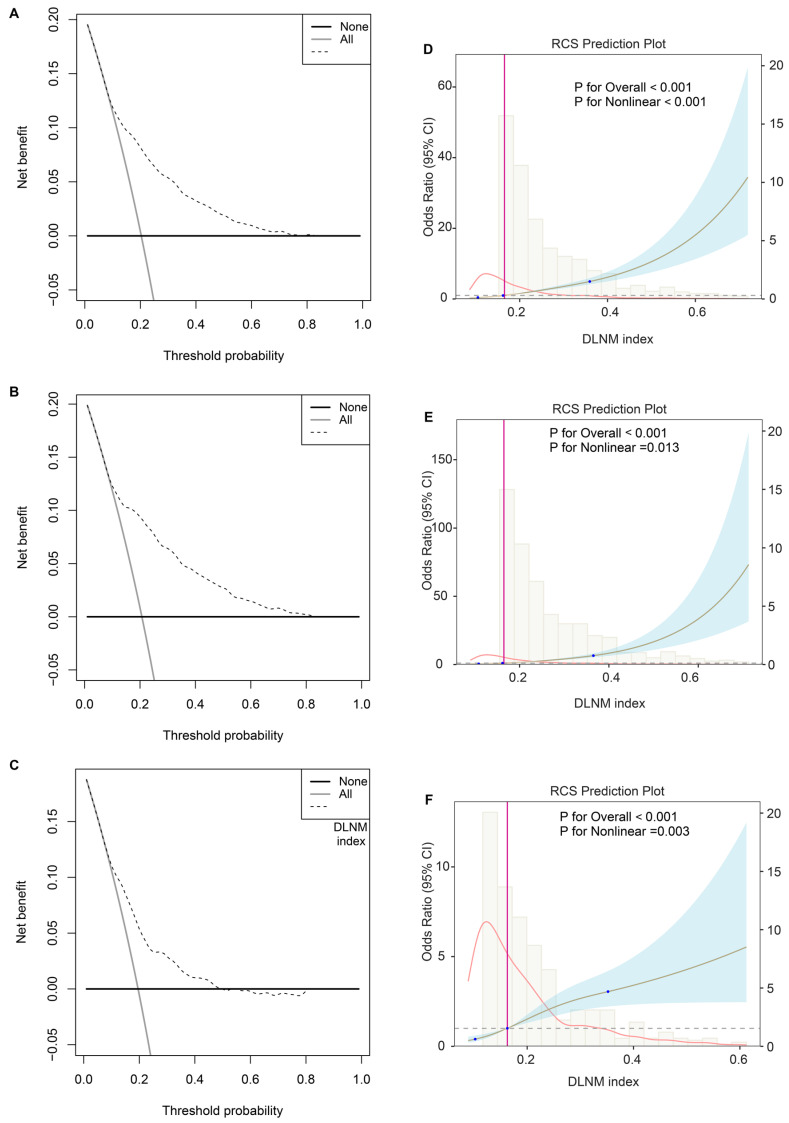
Evaluation of clinical usefulness and nonlinear relationship of DLNM index. (A-C) Decision curve analysis was applied to evaluate the clinical usefulness of DLNM index in entire cohort (A), training cohort (B), validation cohort (C). The Y-axis represents the net benefit. The black line represents the hypothesis that no patients treatment. The X-axis represents the threshold probability. The threshold probability is where the expected benefit of treatment is equal to the expected benefit of avoiding treatment. (D-F) Potential nonlinear for the levels of DLNM index with DLNM risk measured by restricted cubic spline regression with 3 knots in entire cohort (D), training cohort (E), validation cohort (F). The brown line and shadow area represent the estimated OR and the 95% CI.

Third, the DCA plot revealed that across threshold probabilities ranging from 0 to 1, utilising the DLNM index (predictive model) yielded superior net benefits compared to strategies of universal intervention or non-intervention across all three cohorts (**Figure 4A-C**). RCS regression uncovered that the significant nonlinear associations between the DLNM index and DLNM (*P* for nonlinearity < 0.001 in the entire cohort; *P* for nonlinearity = 0.013 in the training cohort; *P* for nonlinearity = 0.003 in the validation cohort) (**Figure 4D-F**). As the DLNM index increased, the odds ratio (OR) of DLNM dramatically increased.

Fourth, to further assess the stability of the model, we performed bootstrap and 10-fold cross-validation in the entire cohort. As a result, ROC, calibration curve, and DCA analysis found that the predictive power of the DLNM index was consistently superior across 10 different cohorts through bootstrap cross-validation (**Figure S1A-C**). Similar results were observed for 10-fold cross-validation (**Figure S1D-F**). Additionally, the accuracy, prevalence, recall, F1-score, MCC, precision, specificity, FNR, and FPR were used to evaluate the DLNM index in 10 different cohorts (**Supplementary material 5**), revealing that the DLNM index is generally stability.

### MLR, subgroup analysis and interaction effect

Univariable and MLR analyses were executed to determine whether the DLNM index remains an independent risk factor for DLNM, regardless of other clinicopathological features. Univariate logistic regression analysis indicated a positive correlation between DLNM index and DLNM in patients PTC according to in the crude model (no covariate was adjusted), with an OR and 95%CI of 2.19 (1.98, 2.42) (*P* < 0.001), with an OR and 95%CI of 2.49 (2.19, 2.83) (*P* < 0.001), with an OR and 95%CI of 1.69 (1.44, 1.99) (*P* < 0.001) (**Table 3**). After adjusting for all covariates, multivariate multivariable regression analyses suggested that DLNM index was an independent predictor of DLNM risk, with an OR and 95%CI of 3.04 (2.48, 3.72) (*P* < 0.001), with an OR and 95%CI of 5.18 (3.90, 6.89) (*P* < 0.001), with an OR and 95%CI of 1.37 (1.07, 1.76) (*P* = 0.014).

To examine potential differences among distinct populations, we performed logistic regression evaluations across multiple subgroups. The analysis demonstrated a notable positive link between the DLNM index and DLNM across all subgroups (*P* < 0.05), except for several small sample size subgroups (pM positive, n=45; superior mediastinal metastasis, n=17) (**Table 4**), revealed that DLNM index is generally robust. Interaction tests revealed significant interactions between DLNM index and PTC subtype (*P* = 0.036 for interaction), small foci of squamous lesions (*P* = 0.021 for interaction), pTNM stage (*P* = 0.023 for interaction), vascular invasion (*P* = 0.01 for interaction). The positive association between DLNM index and DLNM appeared stronger in patients with classical PTC (OR: 2.25; 95 % CI: 2.03-2.5; *P* < 0.001) than follicular PTC (OR: 1.31; 95 % CI: 0.8-2.15; *P* = 0.288).

### Comparison of optimised DLNM index to other models

**Figure 5:**
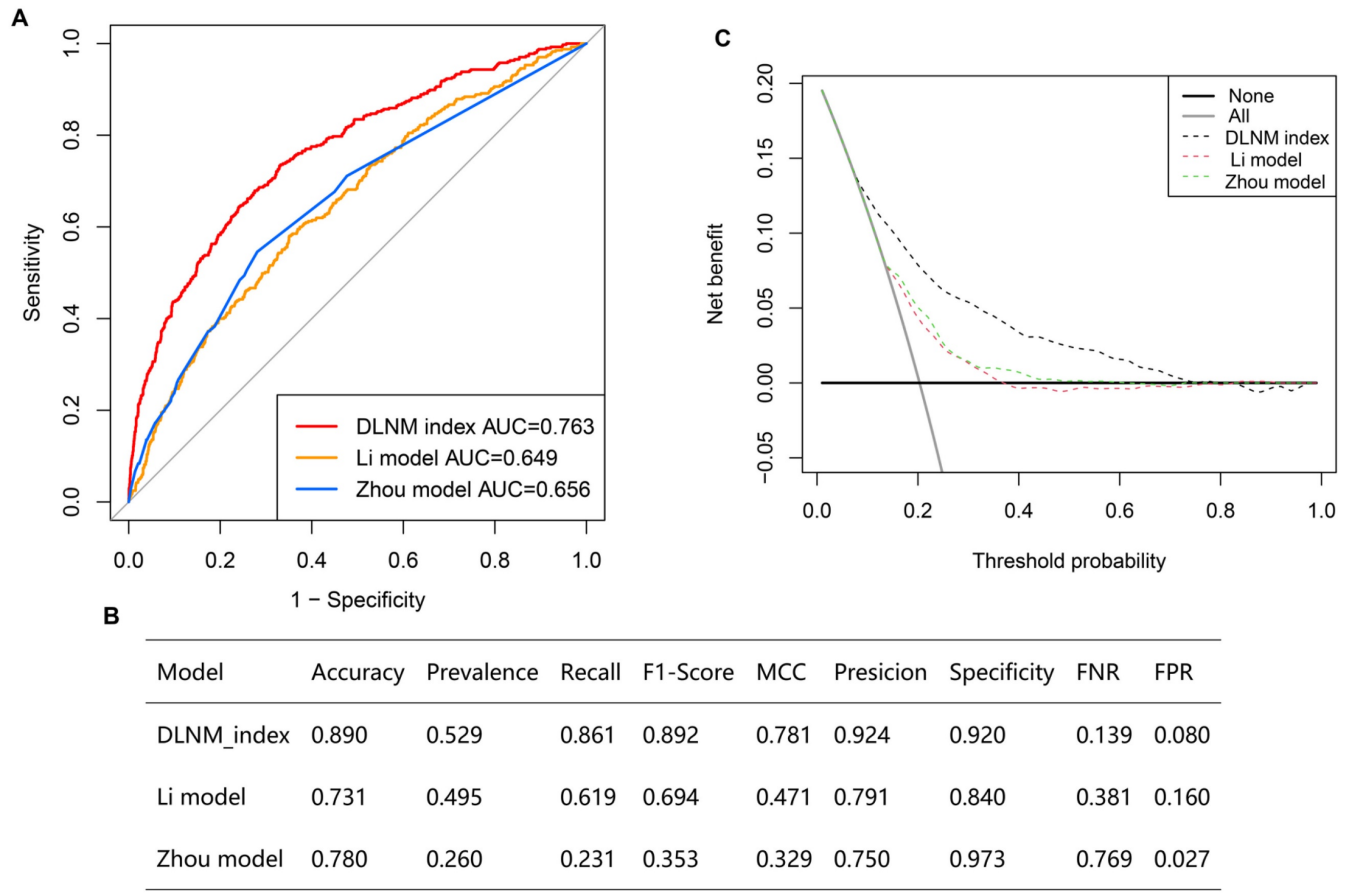
Comparison of optimized DLNM index to other models. (A) Multiple ROC analysis was performed to compare the diagnostic performance of the DLNM index against Li model 11 and Zhou model 12. (B) The model's predictive performance was compared through a comprehensive array of metrics including accuracy, prevalence, recall, F1-score, Matthews correlation coefficient (MCC), precision, specificity, false negative rate (FNR), false positive rate (FPR). (C): Decision curve analysis was applied to evaluate the clinical usefulness of DLNM index against Li model 11 and Zhou model 12. The Y-axis represents the net benefit. The black line represents the hypothesis that no patients die. The Xaxis represents the threshold probability. The threshold probability is where the expected benefit of treatment is equal to the expected benefit of avoiding treatment.

Multiple ROC analyses found the DLNM index performed better in predicting DLNM than the Li model 11 (AUC = 0.649) and the Zhou model 12 (AUC = 0.656) (**Figure 5A**) (*P* < 0.05). Additionally, the accuracy, prevalence, recall, F1-score, MCC, precision, specificity, FNR, and FPR of the DLNM model are superior to the Li model and Zhou model (**Figure 5B**). Notably, the DCA plot demonstrates that the DLNM index exhibits superior performance versus Li's approach or Zhou's framework, as evidenced by the consistency of mortality thresholds (x-axis) and the stratification advantages in risk assessment (y-axis) (**Figure 5C**).

### Model interpretation

**Figure 6:**
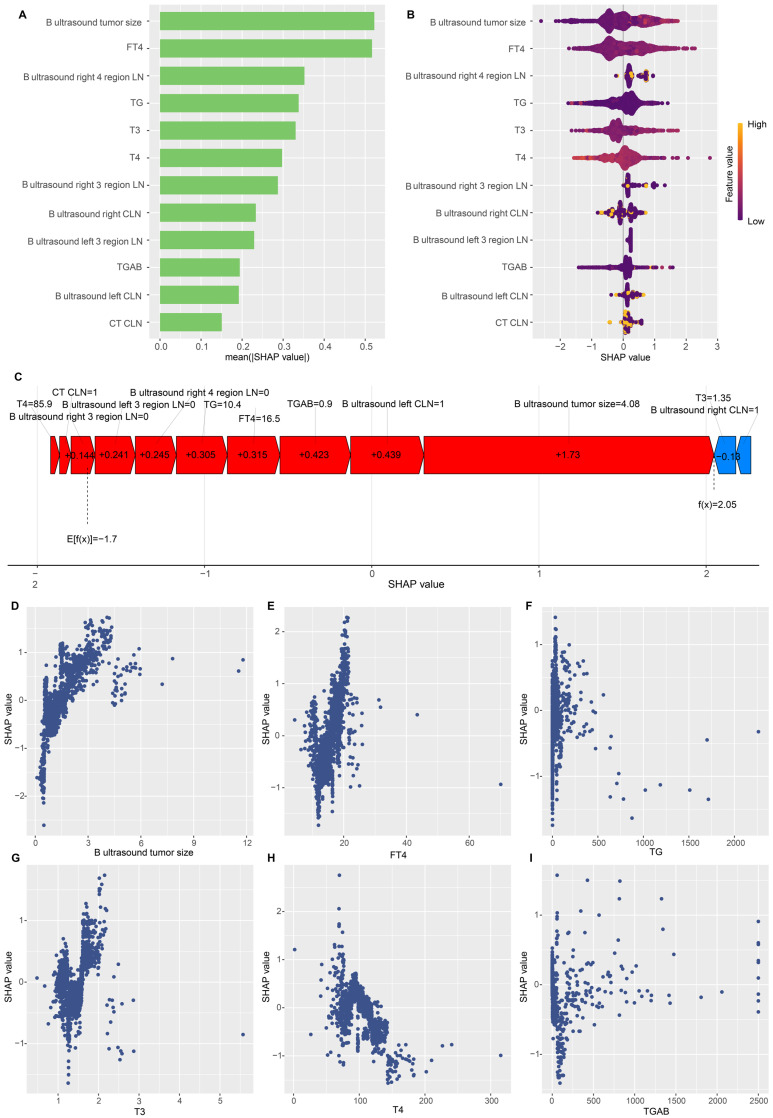
Global and local model explanation by the SHAP method. (A) Summary plot showed the 12 features ranking by mean absolute SHAP values. (B) Each variable name is shown on the left-hand side with the variable with the greatest contribution listed at the top. To the right of the variables, there are colored lines, which are individual points that correspond to observations in the population. A higher value for the variable is represented in yellow, while a lower value for the variable will be shown in purple. A value farther to the right (ie, a higher SHAP value) indicates that the variable is contributed to a prediction of a positive target, such as DLNM. (C) An example of risk factor analysis for a patient with PTC which represented the individual PTC towards the “DLNM” class. (D-I) One-way SHAP dependence plot of the 6 important predictors (continuous variable). (D) B ultrasound tumor size. (E) FT4. (F) TG. (G) T3. (H) T4. (I) TGAB. Each dependence plot shows how a single feature affects the output of the prediction model, and each dot represents a single patient. Specifically, the values of the predictor are represented by the x-axis, and its SHAP values are represented by the y-axis. To interpret these plots, for example, in (D), patients with higher tumor size (as x-axis increased) were associated with a higher SHAP value, which indicated a higher likelihood of DLNM (y-axis also increased).

To gain a thorough insight into the chosen parameters, we utilised the SHAP methodology to emphasise their predictive significance in the optimised DLNM framework. The comparative significance and impact of the leading 12 characteristics on the DLNM framework are depicted in **Figure 6A**, derived through the SHAP methodology's interpretation of the DLNM framework predictions. The analysis identified tumour dimensions as the primary determinant for the prediction framework, succeeded by FT4, right 4 region LN, TG, T3, T4, right 3 regions LN, right CLN, left 3 regions LN, TGAB, left CLN and CT CLN metastasis. **Figure 6B** provides a visual representation of the scope and patterns of these 12 essential characteristics regarding framework effectiveness. Individual characteristic impacts are depicted through distinct colour points: yellow signifies elevated risk values, and purple indicates reduced ones. The positively correlated characteristics encompass tumour dimensions and FT4 (elevated values of these characteristics corresponded to an increased likelihood of DLNM development in PTC patients). Notably, characteristics can influence predictions bidirectionally (enhancing or diminishing DLNM) for patients with varying characteristics, distinguishing it from earlier frameworks (such as the Li model 11 and Zhou model 12), where a specific characteristic value's influence on prediction remains constant.

To enhance our understanding of the optimised DLNM model, we initially analysed patient-specific risk predictions and their risk origins identified through SHAP values. Examining the case with the highest predicted SHAP value (specifically 2.05), we found that enhanced tumour dimensions (4.08 cm, SHAP value = 1.73), suspected left CLN metastasis (SHAP value = 0.439), FT4 levels (16.5 pmol/L, SHAP value = 0.315), and TG measurements (10.4 ng/ml, SHAP value = 0.305) constituted the primary risk factors contributing to the elevated SHAP value **(Figure 6C)**. Furthermore, partial dependence plots were generated for the 6 continuous predictors **(Figure 6D-I)**. These plots provided a visual representation of the comprehensive relationship between features and risk distribution. The plots revealed distinct linear or nonlinear correlations between tumour size, FT4, T3, TG and SHAP value. In particular, DLNM risk probability increased substantially when tumour size, FT4, T3, and TG exceeded specific threshold values.

### Associations between the DLNM index, as well as predictive variables and DLNM

**Figure 7:**
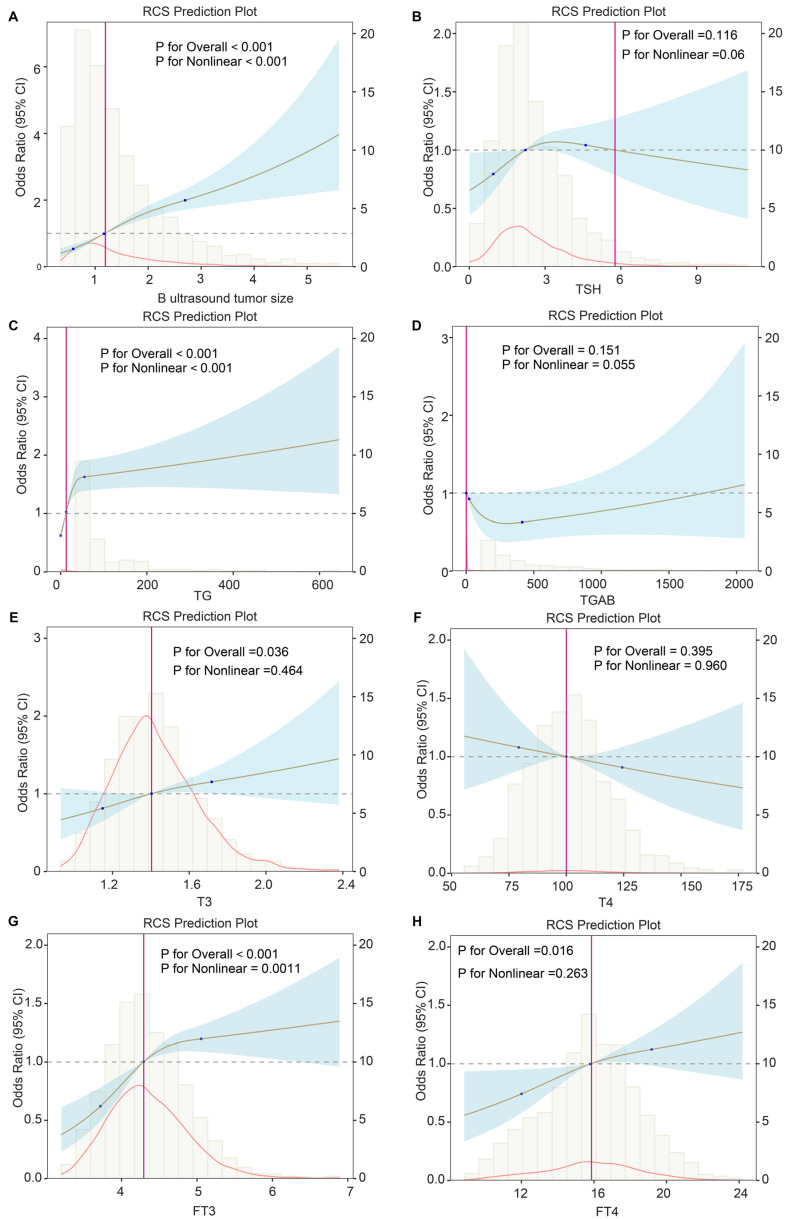
Potential nonlinear for the levels of continuous predictors with DLNM risk measured by restricted cubic spline regression with 3 knots. (A) B ultrasound tumor size. (B) TSH. (C) TG. (D) TGAB. (E) FT3. (F) FT4. (G) T3. (H) T4. The brown line and shadow area represent the estimated OR and the 95% CI. TSH, thyroid stimulating hormone; TG, thyroglobulin; TGAB, anti-thyroglobulin antibodies; T3, triiodothyronine; T4, thyroxine; FT3, free triiodothyronine; FT4, free thyroxine.

To further explore the importance of variables in the predictive model, we performed univariable logistic and RCS regression to investigate the linear and nonlinear associations. We observed 11 and 3 predictors to have linear and nonlinear associations with DLNM risk, respectively (all *P* < 0.013) (**Table 5**). We visualised the corresponding results for continuous variables (**Figure 7**). Tumour size (*P* for linearity < 0.001, *P* for nonlinearity < 0.001), TG (*P* for linearity = 0.003, *P* for nonlinearity < 0.001), and FT3 (*P* for linearity = 0.003, *P* for nonlinearity = 0.0011) showed strong nonlinear relationships with DLNM risk. However, FT4 (*P* for linearity = 0.008, *P* for nonlinearity = 0.263) and T3 (*P* for linearity = 0.013, *P* for nonlinearity = 0.464) showed significant linear but no significant nonlinear associations with DLNM risk.

### Comparison of pathological characteristics between DLNM and non-DLNM cohorts

We compared 25 postoperative pathological features between the DLNM cohort and the non-DLNM cohort in the entire cohort, training cohort, and validation cohort (**Table 6**). It was observed that the tumour size, classical PTC subtype, small foci of squamous lesions, multifocal, vascular invasion, intra-glandular dissemination, capsular invasion, pT stage, and pN stage were higher in the DLNM cohort (all *P* < 0.043) compared with non-DLNM cohort.

## Discussion

Currently, PTC lacks an optimised, explainable ML model to predict DLNM and guide surgical intervention. This study recruited 1993 consecutive individuals with PTC who received curative surgery to develop and validate a robust and generalisable model using preoperative routine clinical indicators. Through feature selection of the Boruta algorithm and 113 permutations of 12 ML methodologies, we established and confirmed a consensus ML-derived diagnostic model (DLNM index) per the higher average AUC (0.763), least variable and more stability from two cohorts. DLNM index exhibited excellent prediction performance, discriminative ability and clinical usefulness, serving as an independent risk factor (*P* < 0.001) regardless of other clinical characteristics. Bootstrapping and 10-fold cross validation and subgroup analysis showed that the DLNM index was generally robust and generalisable. Importantly, it performed better in predicting performance and yielded a larger net benefit than the published model (*P* < 0.05). SHAP interprets the feature importance ranking (tumour size, right 4 regions LN, FT4, TG and T3) and visualises the effect of individual features on the model output as well as the prediction of DLNM by all features. From partial dependence plots, the linear or nonlinear associations between tumour size, FT4, T3, TG and SHAP value can be clearly observed. RCS regression further suggested a nonlinear link between the DLNM index, TG, tumour size, FT3 and DLNM risk. With the development of endoscopic thyroidectomy, the development of transoral vestibular approach, transchin approach, transaxillary approach, subclavian approach and transthoracic and breast approach has increased. However, DLN dissection is difficult in these endoscopic thyroidectomy, and surgeons often neglect DLN dissection. We constructed a model of DLNM based on the patient's preoperative routine clinical examinations (medical history, thyroid function, B-ultrasound, and CT), and preoperatively searched out patients at high risk of DLNM, so as to avoid as much as possible the omission of intraoperative DLN dissection. In short, the DLNM index provides a low-cost, easily accessible, explainable and accurate diagnostic tool for DLNM in PTC with potential clinical applications.

In this retrospective study, we performed the largest current sample size cohort study to investigate risk factors, modelling, and medical implications of DLNM in individuals with PTC. We found that the metastasis rate of the DLN was 20.32% in PTC, which is consistent with previous reports of DLNM rates of 8% to 28% 11,12,22,23. Reports have indicated that DLNM is a marker of tumour aggressiveness and unfavourable outcomes in PTC 8,10. In our study, the DLNM was correlated to larger tumour size, classical PTC subtype, small foci of squamous lesions, multifocal vascular invasion, intra-glandular dissemination, capsular invasion, higher pT stage, and higher pN stage. Hence, the surgeon needs to focus more on the DLNM risk and make individualised operative interventions in PTC. In the era of precision medicine, a low-cost, easily accessible, explainable and accurate DLNM evaluation model will be of clinical significance.

To optimise the functionality and implementation of the DLNM model, the Boruta algorithm examined 28 distinct preoperative parameters to remove unnecessary variables and establish a streamlined, precise variable set. The main advantage of Boruta's algorithm is that it automatically performs feature selection on the dataset without the need to select the set of features that minimise the model cost function for a particular model. In addition, Boruta's algorithm helps us to understand the influences of the dependent variable in a more comprehensive way so that we can perform feature selection better and more efficiently 24, 25. Through the application of Boruta algorithm screening, fourteen independent parameters were ultimately identified for DLNM model construction.

A crucial element enhancing the generalizability and robustness of our prediction model is our proposed novel LOOCV framework that incorporated 12 ML algorithms, resulting in 113 combinations to construct a consensus prediction model. This investigation enrolled a large number of PTC population to improve the statistical power to ensure the reliability and accuracy of the model, which was split into a training cohort (n = 1395) and a validation cohort (n = 598) according to the time to surgery. Additionally, prior studies showed researchers typically selected modelling algorithms based on personal preference and expertise 11, 12. To address this limitation, we utilised 12 ML algorithms suitable for developing an enhanced diagnostic model. We combined these into 113 algorithmic configurations, implementing variable screening and dimensional reduction through Lasso, RF, Stepglm, and glmBoost. Significantly, overfitting remains a challenging issue in AI and ML biomedical modelling, where models often demonstrate strong performance in training sets but underperform in external validation 26. We note that the “Lasso+RF”, “RF”, “glmBoost + RF”, “Stepglm[both]+RF”, and “Stepglm[backward] + RF” models fit well in the training dataset, but failed during validation testing. Hence, the optimal model was Lasso + GBM with a higher average AUC (0.763), minimal variables and better stability from the two cohorts. A final 12-variables prediction model, the DLNM index, was formed using GBM after Lasso minimised redundant data, with the absence of significant multi-collinearity concerns. Encouragingly, through ROC, calibration curve, and DCA analysis, the DLNM index exhibited excellent predictive performance, fitting ability and clinical application value. After adjusting for all covariates, DLNM index remains an independent predictor of DLNM risk in three PTC cohorts. We executed two methods of cross-validation, namely bootstrap and 10-fold cross-validation, in the entire cohort and found that the predictive power of the DLNM index was consistently superior across 10 different cohorts, indicating the stability of the model. Furthermore, our analysis examined the DLNM model's effectiveness across various subgroups through interaction testing to identify potential dataset biases. Consistent subgroup outcomes validated the DLNM model's extensibility and stability. Notably, we revealed a significant interaction between the DLNM index and the PTC subtype. DLNM index can effectively predict DLNM index in classical PTC (OR: 2.25; 95 % CI: 2.03-2.5; *P* < 0.001) but not in follicular PTC (OR: 1.31; 95 % CI: 0.8-2.15; *P* = 0.288). This is consistent with the results of descriptive statistical analysis that the follicular subtype of PTC is less susceptible to DLNM (10.26% *vs* 3,21%; *P* < 0.001) as well as published studies 27, 28.

Two studies also established predictive and diagnostic models to predict DLNM in patients with PTC based on clinicopathological characteristics 11, 12. Therefore, we directly compare the DLNM index to the Li model and Zhou model. The DLNM index (AUC = 0.763) demonstrated outstanding predictive and diagnostic performance than the Li model (AUC = 0.649) and the Zhou model (AUC = 0. 656). A range of other model performance evaluation metrics, including accuracy, prevalence, recall, F1-score, MCC, precision, specificity, FNR, and FPR, also observed similar results. According to the DCA analysis, clinical interventions directed by the DLNM index demonstrated superior net advantages compared to those utilising the Li model or Zhou model approaches. This outcome can potentially be attributed to the following factors: 1) Large sample size (our study n=1993 *vs* Li model n = 581 and Zhou model n = 596) improves precision, greater statistical efficacy, better generalizability, reduces the effect of outliers. 2) The introduction of Boruta's algorithm for feature selection and 12 ML algorithms that yielded 113 combinations to construct a predictive model can identify important feature variables, produce optimal models, and avoid overfitting problems. 3) Inclusion of biochemical indicators for the first time in DLNM model could potentially explain the mechanism of DLNM. Thus, the DLNM index exhibits the capability to detect DLNM and support medical assessments.

The ML approach has been characterised as an obscure system offering minimal insight into its predictive mechanisms 29. Healthcare practitioners might resist its implementation due to reluctance to base clinical judgments on non-transparent data. Therefore, the comprehensibility of diagnostic algorithms remains essential for physician confidence and reliability. Initially, we implemented the SHAP methodology 19, 20, 30 to illuminate the internal workings of DLNM frameworks in forecasting the DLNM of PTC. The SHAP technique enables model interpretation through comprehensive analysis depicting overall system behaviour and specific case examination detailing individual PTC predictions using personalised information. Our investigation's SHAP evaluation identifies variable significance, with neoplasm dimensions emerging as the primary determinant in the DLNM framework, succeeded by FT4, right 4 region LN, TG, T3, and T4. This is consistent with the discovery that tumour size and TG are the two most important variables in the initial feature variable selection through Boruta's arithmetic. Additionally, 1-way SHAP dependence plots visually displayed the global link between feature and risk distribution. From these plots, we clearly noted the linear or nonlinear links between tumour size, FT4, T3, TG and SHAP value. Specifically, when tumour size, FT4, T3, and TG are above a certain threshold, the likelihood of developing DLNM increases dramatically. This patient-specific interpretable framework enables healthcare providers to synthesise predictions and explanations with clinical expertise for enhanced decision-making.

To elucidate how variables function within the DLNM model and provide enhanced clinical guidance, we systematically analysed both linear and nonlinear associations between the DLNM index as well as predictive variables and DLNM risk through logistic and RCS regression analysis. As a result, tumour size, TG, FT3, FT4, and T3 showed strong linear or nonlinear relationships with DLNM risk. A recently published meta-analysis summarising four articles found that the DLN-positive rate in individuals with tumour size >1 cm was 3.55 times higher than DLN-negative (OR = 3.55, 95% CI: 2.34-5.40, *P* < 0.01) 31. Our study further found that tumour size was positively related to the risk of DLNM with the nonlinear association; Boruta's arithmetic and SHAP analysis uncovered that it was found to be one of the most important variables in DLNM models. This suggests DLNM's connection to cancer lesion dimensions. Contemporary research indicates that TG in fine-needle aspiration biopsy extract demonstrates significant diagnostic utility for lateral cervical LNM in PTC cases, showing strong concordance with post-surgical pathological findings 32. Wang *et al.* identified a positive association between FT3 measurements and central LNM in PTC (*P* < 0.001) 33; Diessl *et al.* determined that elevated FT3 correlates with poorer outcomes in advanced differentiated thyroid cancer 34. Duan *et al.* established a meaningful connection between elevated FT4 and CLNM occurrence 35. Recent investigations increasingly examine thyroid hormone-tumor development relationships 36, 37. Evidence suggests elevated free thyroid hormones might facilitate tumour cell malignant transformation by stimulating crucial signalling pathways, including ERK1/ERK2 and PI3K, enhancing invasiveness, metastasis, and proliferation. Our study first demonstrated a significant linear or nonlinear relationship between TG, FT3, FT4, and T3 with DLNM risk, though additional validation through larger cohorts and molecular mechanism studies remains necessary. Thus, clinicians could initially assess the likelihood of DLNM by these routine preoperative findings (B ultrasound tumour size, TG, FT3, FT4, T3), which have important clinical practice value.

In contrast to prior investigations, our research exhibits several distinctive characteristics. (1) This study develops a prediction model for DLNM prediction in patients with PTC based on preoperative routine clinical features with the largest current sample size cohort. Our predictive model incorporates only preoperative routine testing metrics, unlike published studies that incorporate postoperative metrics, and the model is not effective in guiding the first surgical intervention. (2) By introducing Boruta's feature selection algorithm and 12 ML algorithms, 113 combinations are generated to build consensus prediction models, which can identify important feature variables, produce optimal models, and avoid overfitting problems. (3) The SHAP method was applied to mitigate the concern of the “black-box” issue with an undirect interpretation of the ML technique. SHAP explains the importance of ranking features and visualises global and individual risk prediction. This patient-level interpretable model allows clinicians to combine predictions and explanations with their empirical knowledge to facilitate decision-making. (4) The study design incorporated thyroid hormone indicators, has a potential mechanism to explain LNM, and by logistic and RCS regression analysis, we found for the first time that TG, FT3, FT4, and T3 demonstrated significant positive associations with DLNM in a linear or nonlinear way.

While we endeavoured to conduct our investigation with maximum thoroughness and precision, certain constraints warrant acknowledgement. Initially, this investigation exemplifies retrospective examinations conducted at an individual tertiary cancer facility. However, our DLNM model demonstrated impressive performance in DLNM prediction, such as ROC analysis, calibration curve, Bootstrapping and 10-fold cross validation, RCS regression, MLR, and subgroup analysis. The established framework requires external dataset verification before widespread implementation. Subsequent investigations should encompass multi-institutional, expanded cohort examinations to confirm and broaden these discoveries. Second, the inclusion of preoperative B ultrasound and CT reports in the DLNM model inevitably suffers from the subjectivity of the imaging physician, in addition to the fact that the accuracy of the model may be influenced by the level of the examiner. With the future application of artificial intelligence in image diagnosis, these problems may be avoided. Third, the Chinese guidelines advocate the preventive removal of CLNs for patients with PTC. Hence, these outcomes might not be relevant in areas where surgeons exclusively conduct therapeutic CLN removal for individuals with PTC. Fourth, we revealed for the first time that thyroid hormone indicators (TG, FT3, FT4, T3) had a significant positive correlation with DLNM risk in a linear or nonlinear manner. Additional research at both clinical and molecular levels is essential to elucidate these hormonal relationships and their influence on DLNM development.

## Conclusion

This investigation represents a pioneering effort to develop and validate a robust and generalisable model for DLNM prediction in PTC patients using preoperative routine clinical indicators. We evaluated 113 distinct combinations incorporating 12 ML techniques to construct and authenticate a unified diagnostic approach (DLNM index). SHAP interprets the feature importance ranking and visualises global and individual explanations for the DLNM model. The DLNM index is a low-cost, accessible, interpretable and accurate tool for diagnosing PTC DLNM with potential clinical applications.

## Supplementary Material

Supplementary figures and tables.

## Figures and Tables

**Table 1:** Comparison of baseline characteristics and biochemical indicators between DLNM and non-DLNM groups in the three cohorts.

Clinical parameters	Entire cohort (n=1993)			Training cohort (n = 1395)			Validation cohort (n = 598)		
	Non-DLNM (n=1588)	DLNM (n=405)	*P*-value	Non-DLNM (n=1107)	DLNM (n=288)	*P*-value	Non-DLNM (n=481)	DLNM (n=117)	*P*-value
Male sex, No. (%)	451 (28.40)	119 (29.38)	0.742	325 (29.36)	81 (28.12)	0.736	126 (26.20)	38 (32.48)	0.211
Age, median (IQR), year	37 (18-76)	38 (21-78)	0.011*	37 (18-76)	39 (21-78)	0.065	34 (20-84)	36 (24-69)	0.081
Time to diagnose the tumor, median (IQR), day	60 (1-12775)	60 (1-3650)	0.016*	60 (1-12775)	60 (1-3285)	0.021*	60 (1-4746)	60 (3-3650)	0.410
B ultrasound report									
Tumor size, median (IQR), cm	1.1 (0.1-7.8)	1.53 (0.38-11.8)	<0.001*	1.14 (0.1-7.21)	1.5 (0.38-11.8)	<0.001*	1.02 (0.38-7.8)	1.64 (0.5-11.55)	<0.001*
Tumor site, upper, No. (%)	151 (9.51)	27 (6.67)	0.078	105 (9.49)	15 (5.21)	0.043*	46 (9.56)	12 (10.26)	0.239
Tumor site, middle, No. (%)	179 (11.27)	35 (8.64)		124 (11.20)	30 (10.42)		55 (11.43)	5 (4.27)	
Tumor site, below, No. (%)	631 (39.74)	170 (41.98)		436 (39.39)	120 (41.67)		195 (40.54)	50 (42.74)	
Tumor site, isthmus, No. (%)	98 (6.17)	35 (8.64)		61 (5.51)	26 (9.03)		37 (7.69)	9 (7.69)	
Tumor site, multiple sites, No. (%)	529 (33.31)	138 (34.07)		529 (33.31)	97 (33.68)		148 (30.77)	41 (35.04)	
Bilateral tumor, No. (%)	391 (24.62)	109 (26.91)	0.376	276 (24.93)	76 (26.39)	0.667	115 (23.91)	33 (28.21)	0.397
Close to anterior capsule, No. (%)	142 (8.94)	28 (6.91)	0.228	101 (9.12)	25 (8.68)	0.906	41 (8.52)	3 (2.56)	0.044*
Close to posterior capsule, No. (%)	171 (10.77)	42 (10.37)	0.888	107 (9.67)	31 (10.76)	0.656	64 (13.31)	11 (9.40)	0.323
Close to trachea, No. (%)	62 (3.90)	8 (1.98)	0.083	46 (4.16)	6 (2.08)	0.139	16 (3.33)	2 (1.71)	0.538
Left CLN suspicious metastasis, No. (%)	277 (17.44)	132 (32.59)	<0.001*	191 (17.25)	91 (31.60)	<0.001*	86 (17.88)	41 (35.04)	<0.001*
Left 2 region LN suspicious metastasis, No. (%)	32 (2.02)	15 (3.70)	0.069	27 (2.44)	10 (3.47)	0.444	5 (1.04)	5 (4.27)	0.041
Left 3 region LN suspicious metastasis, No. (%)	77 (4.85)	49 (12.10)	<0.001*	57 (5.15)	34 (11.81)	<0.001*	20 (4.16)	15 (12.82)	0.001*
Left 4 region LN suspicious metastasis, No. (%)	181 (11.40)	86 (21.23)	<0.001*	120 (10.84)	57 (19.79)	<0.001*	61 (12.68)	29 (24.79)	0.002*
Right CLN suspicious metastasis, No. (%)	278 (17.51)	139 (34.32)	<0.001*	204 (18.43)	103 (35.76)	<0.001*	74 (15.38)	36 (30.77)	<0.001*
Right 2 region LN suspicious metastasis, No. (%)	38 (2.39)	27 (6.67)	<0.001*	24 (2.17)	17 (5.90)	0.002*	14 (2.91)	10 (8.55)	0.012*
Right 3 region LN suspicious metastasis, No. (%)	78 (4.91)	59 (14.57)	<0.001*	51 (4.61)	45 (15.62)	<0.001*	27 (5.61)	14 (11.97)	0.014*
Right 4 region LN suspicious metastasis, No. (%)	179 (11.27)	104 (25.68)	<0.001*	126 (11.38)	76 (26.39)	<0.001*	53 (11.02)	28 (23.93)	<0.001*
CT report									
CLN suspicious metastasis, No. (%)	615 (38.73)	236 (58.27)	<0.001*	439 (39.66)	168 (58.33)	<0.001*	176 (36.59)	68 (58.12)	<0.001*
Lateral cervical LN suspicious metastasis, No. (%)	697 (43.89)	247 (60.99)	<0.001*	488 (44.08)	183 (63.54)	<0.001*	209 (43.45)	64 (54.70)	<0.037*
Superior mediastinum LN suspicious metastasis, No. (%)	36 (2.27)	8 (1.98)	0.867	29 (2.62)	6 (2.08)	0.759	7 (1.46)	2 (1.71)	1.000
Lung suspicious metastasis, No. (%)	42 (2.64)	4 (0.99)	0.072	31 (2.80)	3 (1.04)	0.131	11 (2.29)	1 (0.85)	0.533
TSH, median (IQR), mIU/L	2.176 (0.005-30.072)	2.299 (0.005-16.363)	0.396	2.176 (0.005-30.072)	2.269 (0.009-16.363)	0.601	2.179 (0.005-17.235)	2.435 (0.005-13.905)	0.450
TG, median (IQR), ng/ml	11.5 (0.1-1699.56)	16.7 (0.12-2263.87)	<0.001*	11.5 (0.1-1699.56)	16.225 (0.17-2263.87)	0.006*	11.5 (0.1-1509.57)	18.38 (0.12-1713.99)	0.027*
TGAB, median (IQR), IU/ml	0.9 (0.9-2500)	0.9 (0.9-2500)	0.690	0.9 (0.9-2500)	0.9 (0.9-2500)	0.655	0.9 (0.9-2500)	0.9 (0.9-460.5)	0.084
T3, median (IQR), nmol/L	1.39 (0.48-5.57)	1.42 (0.76-2.49)	0.011*	1.39 (0.67-5.57)	1.42 (0.76-2.17)	0.080	1.39 (0.48-2.38)	1.43 (0.92-2.49)	0.037*
T4, median (IQR), nmol/L	100.35 (26.3-315.25)	99.86 (1.78-226.55)	0.172	100.3 (26.3-240.63)	100.08 (41.9-226.55)	0.613	101.4 (42.9-315.25)	99.59 (1.78-160.11)	0.113
FT3, median (IQR), pmol/L	4.28 (2.31-24.29)	4.49 (2.88-109.38)	0.003*	4.28 (3.08-24.29)	4.455 (3.23-6.19)	0.009*	4.28 (2.31-7.42)	4.53 (2.88-109.38)	0.017*
FT4, median (IQR), pmol/L	15.735 (6.39-70.06)	16.28 (4.39-31.75)	0.005*	15.7 (7.52-70.06)	16.24 (8.85-23.36)	0.010*	15.8 (6.39-25.96)	16.32 (4.39-31.75)	0.261

Abbreviations: No., number; DLNM, delphian lymph node metastasis; LN, lymph node; CLN, central lymph node; IQR, interquartile range; CT, computed tomography; TSH, thyroid stimulating hormone; TG, thyroglobulin; TGAB, anti-thyroglobulin antibodies; T3, triiodothyronine; T4, thyroxine; FT3, free triiodothyronine; FT4, free thyroxine. *P < 0.05.

**Table 2:** Predictive performance of the DLNM index in the three cohorts.

Cohort	Accuracy	Prevalence	Recall	F1-Score	MCC	Presicion	Specificity	FNR	FPR
Entire cohort	0.890	0.529	0.861	0.892	0.781	0.924	0.920	0.139	0.080
Training cohort	0.918	0.531	0.892	0.920	0.837	0.950	0.947	0.108	0.053
Validation cohort	0.846	0.518	0.771	0.839	0.703	0.918	0.926	0.229	0.074

Abbreviations: DLNM, delphian lymph node metastasis; MCC, Matthews correlation coefficient; FNR, false negative rate; FPR, false positive rate

**Table 3:** Univariable and multivariable logistic regression analysis for prediction of DLNM.

	Training cohort		Validation cohort		Entire cohort	
	OR (95 % CI)	*P*-value	OR (95 % CI)	*P*-value	OR (95 % CI)	*P*-value
Coarse model	2.49 (2.19-2.83)	< 0.001*	1.69 (1.44-1.99)	< 0.001*	2.19 (1.98-2.42)	< 0.001*
Model 1	2.50 (2.20-2.84)	< 0.001*	1.67 (1.42-1.96)	< 0.001*	2.18 (1.97-2.41)	< 0.001*
Model 2	2.71 (2.32-3.16)	< 0.001*	1.67 (1.32-2.06)	< 0.001*	2.29 (2.04-2.57)	< 0.001*
Model 3	5.18 (3.90-6.89)	< 0.001*	1.37 (1.07-1.76)	0.014*	3.04 (2.48-3.72)	< 0.001*

Coarse model, no covariate was adjusted. Model 1, Sex, age, and time to diagnose the tumor were adjusted. Model 2, Sex, age, time to diagnose the tumor, B ultrasound tumor location, B ultrasound bilateral tumor, B ultrasound close to anterior capsule, B ultrasound close to posterior capsule, B ultrasound close to trachea, B ultrasound left 2 region LN, B ultrasound left 4 region LN, B ultrasound right 2 region LN, CT lateral cervical LN, CT superior mediastinum LN, CT lung metastasis, TSH, and FT3 were adjusted. Model 3, Sex, age, time to diagnose the tumor, B ultrasound tumor size, B ultrasound tumor location, B ultrasound bilateral tumor, B ultrasound close to anterior capsule, B ultrasound close to posterior capsule, B ultrasound close to trachea, B ultrasound left CLN, B ultrasound left 2 region LN, B ultrasound left 3 region LN,B ultrasound left 4 region LN, B ultrasound right CLN, B ultrasound right 2 region LN, B ultrasound right 3 region LN, B ultrasound right 4 region LN, CT CLN, CT lateral cervical LN, CT superior mediastinum LN, CT lung metastasis, TSH, TG, TGAB, T3, T4, FT3, and FT4 were adjusted. Abbreviations: DLNM, delphian lymph node metastasis; LN, lymph node; CLN, central lymph node; OR, odds ratio; CI, confidence interval; CT, computed tomography; TSH, thyroid stimulating hormone; TG, thyroglobulin; TGAB, anti-thyroglobulin antibodies; T3, triiodothyronine; T4, thyroxine; FT3, free triiodothyronine; FT4, free thyroxine. *P < 0.05.

**Table 4:** Subgroup analysis for the correlation between the DLNM index and the risk of DLNM in entire cohort.

Variable	Count	Percent	OR	Lower	Upper	*P* value	*P* for interaction
Overall	1993	100	2.19	1.98	2.42	<0.001	
Sex							0.06
Female	1423	71.4	2.36	2.07	2.67	<0.001	
Male	570	28.6	1.94	1.65	2.27	<0.001	
Age, year							0.952
≤ 36	1000	50.2	2.17	1.87	2.53	<0.001	
> 36	993	49.8	2.19	1.91	2.5	<0.001	
Time, day							0.068
≤ 60	1123	56.3	2.04	1.81	2.3	<0.001	
> 60	870	43.7	2.48	2.08	2.95	<0.001	
PTC subtype							0.036
Follicular	176	8.8	1.31	0.8	2.15	0.288	
Classical	1817	91.2	2.25	2.03	2.5	<0.001	
Fibrosis							0.4
No	1476	74.1	2.13	1.91	2.38	<0.001	
Yes	517	25.9	2.37	1.91	2.94	<0.001	
Small foci of squamous lesions							0.021
No	1893	95	2.24	2.02	2.49	<0.001	
Yes	100	5	1.46	1.02	2.07	0.036	
Hashimoto thyroiditis							0.763
No	1557	78.1	2.2	1.97	2.45	<0.001	
Yes	436	21.9	2.11	1.63	2.72	<0.001	
pT stage							0.119
T1	1100	55.2	2.45	1.99	3.03	<0.001	
T2-T4	893	44.8	2.02	1.78	2.29	<0.001	
pN stage							NA
Negative	571	28.7	NA	NA	NA	NA	
Positive	1422	71.3	1.87	1.69	2.07	<0.001	
pM stage							0.131
No	1948	97.7	2.23	2.02	2.47	<0.001	
Yes	45	2.3	1.36	0.72	2.57	0.344	
pTNM stage							0.023
I	1788	89.7	2.29	2.05	2.55	<0.001	
II-IV	205	10.3	1.67	1.31	2.14	<0.001	
Tumor size, cm							0.22
≤ 1.2	1091	54.7	2.43	1.99	2.96	<0.001	
> 1.2	902	45.3	2.09	1.84	2.38	<0.001	
Multifocal							0.575
No	1248	62.6	2.11	1.85	2.42	<0.001	
Yes	745	37.4	2.24	1.93	2.61	<0.001	
Vascular invasion							0.01
No	1686	84.6	2.29	2.02	2.6	<0.001	
Yes	307	15.4	1.73	1.46	2.06	<0.001	
Intra-glandular dissemination							0.128
No	1937	97.2	2.17	1.96	2.41	<0.001	
Yes	56	2.8	1.62	1.13	2.33	0.008	
Capsular invasion							0.818
No	469	23.5	2.22	1.69	2.92	<0.001	
Yes	1524	76.5	2.15	1.93	2.39	<0.001	
Extracapsular spread							0.771
No	796	39.9	2.24	1.86	2.7	<0.001	
Yes	1197	60.1	2.17	1.93	2.44	<0.001	
Trachea invasion							0.44
No	1892	94.9	2.21	1.99	2.45	<0.001	
Yes	101	5.1	1.92	1.36	2.7	<0.001	
Nerve invasion							0.129
No	1840	92.3	2.25	2.02	2.51	<0.001	
Yes	153	7.7	1.79	1.36	2.36	<0.001	
Superior mediastinal metastasis							0.259
No	1976	99.1	2.2	1.99	2.43	<0.001	
Yes	17	0.9	1.47	0.73	2.94	0.278	

Abbreviations: DLNM, delphian lymph node metastasis; LN, lymph node; CLN, central lymph node; PTC, papillary thyroid cancer; NA, not available; IQR, interquartile range; pT, pathological tumor size; pN, pathological node; pM, pathological metastasis; pTNM, pathological tumor node metastasis. *P < 0.05.

**Table 5:** Nonlinear and linear associations between clinical variables and DLNM.

Factors	Subgroup	Linear association		Nonlinear association	
		OR (95%CI)	*P*	Chi-square	*P*
B ultrasound tumor size		1.56 (1.41-1.73)	< 0.001*	78.60	< 0.001*
B ultrasound left CLN	No	1			
	Yes	2.29(1.79-2.92)	< 0.001*	NA	NA
B ultrasound left 3 region LN	No	1			
	Yes	2.70(1.85-3.94)	< 0.001*	NA	NA
B ultrasound right CLN	No	1			
	Yes	2.46(1.93-3.14)	< 0.001*	NA	NA
B ultrasound right 2 region LN	No	1			
	Yes	2.91 (1.76-4.83)	< 0.001*	NA	NA
B ultrasound right 3 region LN	No	1			
	Yes	3.30 (2.31-4.72)	< 0.001*	NA	NA
B ultrasound right 4 region LN	No	1			
	Yes	2.72 (2.07-3.57)	< 0.001*	NA	NA
CT CLN	No	1			
	Yes	2.21 (1.77-2.76)	< 0.001*	NA	NA
TG		1.01 (1.00-1.02)	0.003*	27.96	< 0.001*
TGAB		1.01 (0.99-1.00)	0.691	3.69	0.055
T3		1.67 (1.11-2.51)	0.013*	0.54	0.46
T4		0.99 (0.99-1.02)	0.172	0.002	0.96
FT3		1.29 (1.09-1.53)	0.003*	10.50	0.001*
FT4		1.05 (1.01-1.09)	0.008*	1.26	0.26

Abbreviations: DLNM, delphian lymph node metastasis; LN, lymph node; CLN, central lymph node; OR, odds ratio; CI, confidence interval; CT, computed tomography; TSH, thyroid stimulating hormone; TG, thyroglobulin; TGAB, anti-thyroglobulin antibodies; T3, triiodothyronine; T4, thyroxine; FT3, free triiodothyronine; FT4, free thyroxine. NA, not available; *P < 0.05.

**Table 6:** Comparison of pathological characteristics between DLNM and non-DLNM groups in the three cohorts.

Pathological features	Entire cohort (n=1993)			Training cohort (n = 1395)			Validation cohort (n = 598)		
	Non-DLNM (n=1588)	DINM (n=405)	*P*-value	Non-DLNM (n=1107)	DINM (n=288)	*P*-value	Non-DLNM (n=481)	DINM (n=117)	*P*-value
Tumor size, median (IQR), cm	1.1 (0.1-6.2)	1.5 (0.1-7)	<0.001*	1.1 (0-6)	1.5 (0.1-7)	<0.001*	1 (0.1-6.2)	1.7 (0.3-5.5)	<0.001*
PTC subtype, follicular, No. (%)	163 (10.26)	13 (3.21)	<0.001*	120 (10.84)	11 (3.82)	<0.001*	126 (26.20)	38 (32.48)	<0.001*
Fibrosis, No. (%)	429 (27.02)	88 (21.73)	0.035*	296 (26.74)	59 (20.49)	0.036*	133 (27.65)	29 (24.79)	0.611
Small foci of squamous lesions, No. (%)	68 (4.28)	32 (7.9)	0.0015*	51 (4.61)	20 (6.95)	0.025*	17 (3.53)	12 (10.26)	0.005*
Hashimoto thyroiditis, No. (%)	363 (22.86)	73 (18.02)	0.042*	250 (22.58)	56 (19.44)	0.286	113 (23.49)	17 (14.53)	0.047*
Multifocal, No. (%)	559 (35.20)	186 (45.93)	<0.001*	396 (35.77)	134 (46.53)	0.001*	163 (33.89)	52 (44.44)	0.043*
Vascular invasion, No. (%)	190 (11.96)	117 (28.89)	<0.001*	136 (12.29)	84 (29.17)	<0.001*	54 (11.23)	33 (28.21)	<0.001*
Intra-glandular dissemination, No. (%)	22 (1.39)	34 (8.40)	<0.001*	17 (1.54)	26 (9.03)	<0.001*	5 (1.04)	8 (6.84)	<0.001*
Capsular invasion, No. (%)	1182 (74.43)	342 (84.44)	<0.001*	813 (73.44)	241 (83.68)	<0.001*	369 (76.72)	101 (86.32)	0.032*
Extracapsular spread, No. (%)	931 (58.63)	266 (65.68)	0.011*	645 (58.27)	184 (63.89)	0.096	286 (59.46)	82 (70.09)	0.044*
Trachea invasion, No. (%)	72 (4.53)	29 (7.16)	0.043	51 (4.61)	22 (7.64)	0.056	21 (4.37)	7 (5.98)	0.618
Nerve invasion, No. (%)	116 (7.30)	37 (9.14)	0.258	80 (7.23)	17 (5.90)	0.511	36 (7.48)	20 (17.09)	0.003*
Superior mediastinal metastasis, No. (%)	10 (0.63)	7 (1.73)	0.065	9 (0.81)	5 (1.74)	0.285	1 (0.21)	2 (1.71)	0.183
pT stage, 1a, No. (%)	954 (60.08)	146 (36.05)	<0.001*	656 (59.26)	105 (36.46)	<0.001*	298 (61.95)	41 (35.04)	<0.001*
pT stage, 1b, No. (%)	467 (29.41)	143 (35.31)		332 (29.99)	113 (39.24)		135 (28.07)	30 (25.64)	
pT stage, 2, No. (%)	91 (5.73)	76 (18.77)		70 (6.32)	48 (16.67)		21 (4.37)	28 (23.93)	
pT stage, 3a, No. (%)	25 (1.57)	15 (3.70)		17 (1.54)	11 (3.82)		8 (1.66)	4 (3.42)	
pT stage, 3b, No. (%)	39 (2.46)	17 (4.20)		24 (2.17)	4 (1.39)		15 (3.12)	13 (11.11)	
pT stage, 4a, No. (%)	12 (0.76)	8 (1.98)		8 (0.72)	7 (2.43)		4 (0.83)	1 (0.85)	
pN stage, 0, No. (%)	570 (35.89)	0 (0.00)	<0.001*	394 (35.59)	0 (0.00)	<0.001*	176 (36.59)	0 (0.00)	<0.001*
pN stage, 1a, No. (%)	646 (40.68)	195 (48.15)		447 (40.38)	137 (47.57)		199 (41.37)	58 (49.57)	
pN stage, 1b, No. (%)	372 (23.43)	210 (51.85)		266 (24.03)	151 (52.43)		106 (22.04)	59 (50.43)	
pM stage, No. (%)	41 (2.58)	4 (0.99)	0.082	31 (2.80)	3 (1.04)	0.131	10 (2.08)	1 (0.85)	0.617
pTNM stage, I, No. (%)	1433 (90.24)	355 (87.65)	0.117	989 (89.34)	251 (87.15)	0.197	444 (92.31)	104 (88.89)	0.312
pTNM stage, II, No. (%)	152 (9.57)	48 (11.85)		115 (10.39)	35 (12.15)		37 (7.69)	13 (11.11)	
pTNM stage, III, No. (%)	0 (0.00)	1 (0.25)		0 (0.00)	1 (0.25)		0 (0.00)	0 (0.00)	
pTNM stage, IV, No. (%)	3 (0.19)	1 (0.25)		3 (0.19)	1 (0.25)		0 (0.00)	0 (0.00)	

Abbreviations: No., number; DLNM, delphian lymph node metastasis; PTC, papillary thyroid cancer; LN, lymph node; CLN, central lymph node; IQR, interquartile range; pT, pathological tumor size; pN, pathological node; pM, pathological metastasis; pTNM, pathological tumor node metastasis. *P < 0.05.

## Abbreviations

PTC: papillary thyroid cancer
LNM: lymph node metastasis
ML: machine learning
MR: mendelian randomization
DLNM: delphian lymph node metastasis
RCS: restricted cubic spline
SHAP: Shapley Additive Explanations
CI: confidence interval
OR: odds ratio
SE: standard error
ROC: receiver operating characteristic
AUC: area under curve
Enet: elastic network
LASSO: least absolute shrinkage and selection operator
RF: random forest
GBM: gradient boosting machine
SuperPC: supervised principal components
plsRglm: partial least squares regression for generalized linear models, SVM: support vector machine
LOOCV: leave-one-out cross-validation
DCA: decision curve analysis
LDA: linear discriminant analysis
XGBoost: eXtreme Gradient Boosting
GBM: gradient boosting machine
TRIPOD: Transparent Reporting of a Multivariable Prediction Model forIndividual Prognosis or Diagnosis
CND: central neck dissection
LND: lateral neck dissection
CT: computed tomography
TSH: thyroid stimulating hormone
TG: thyroglobulin
TGAB: anti-thyroglobulin antibodies
T3: triiodothyronine
T4: thyroxine
FT3: free triiodothyronine
FT4: free thyroxine
CLN: paratracheal lymph node
IQR: interquartile range
pT: pathological tumor size
pN: pathological node
pM: pathological metastasis
pTNM: pathological tumor node metastasis
AJCC: American Joint Committee on Cancer. SD: standard deviation
MCC: Matthews correlation coefficient
FNR: false negative rate
FPR: false positive rate
