# Supplementary material for: Development and validation of an explainable machine learning model to predict Delphian lymph node metastasis in papillary thyroid cancer: a large cohort study
Source: J Cancer. 2025 Mar 3;16(6):2041–61. doi: 10.7150/jca.110141 (PMC11905415; doi:10.7150/jca.110141)
Supplement: Supplementary file 1 — Supplementary figures and tables. [file jcav16p2041s1.zip › Figure S1+S2.pdf]

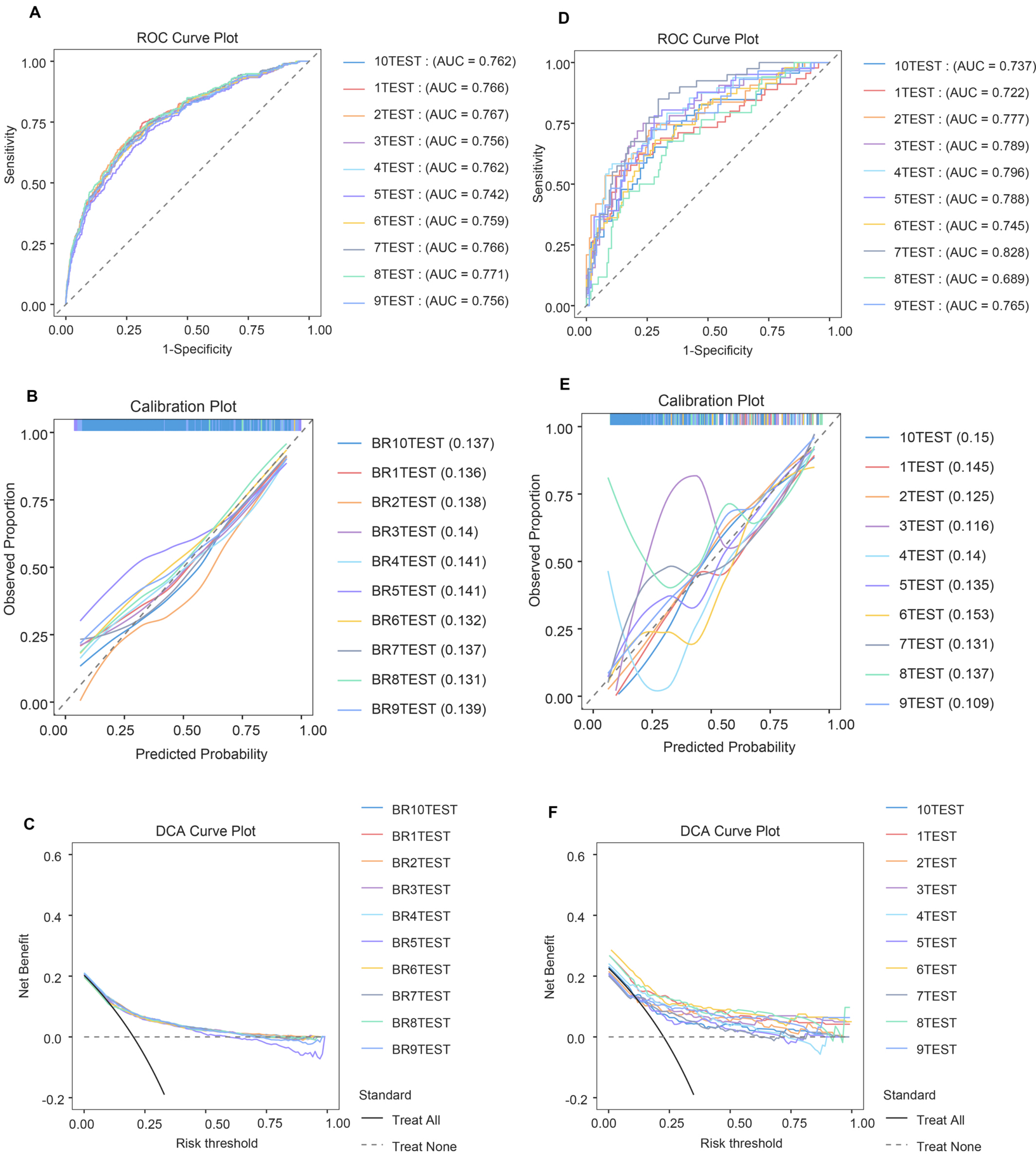
A
ROC Curve Plot
Sensitivity
1-Specificity
0.00
0.25
0.50
0.75
1.00
10TEST : (AUC = 0.762)
1TEST : (AUC = 0.766)
2TEST : (AUC = 0.767)
3TEST : (AUC = 0.756)
4TEST : (AUC = 0.762)
5TEST : (AUC = 0.742)
6TEST : (AUC = 0.759)
7TEST : (AUC = 0.766)
8TEST : (AUC = 0.771)
9TEST : (AUC = 0.756)
D
ROC Curve Plot
Sensitivity
1-Specificity
10TEST : (AUC = 0.737)
1TEST : (AUC = 0.722)
2TEST : (AUC = 0.777)
3TEST : (AUC = 0.789)
4TEST : (AUC = 0.796)
5TEST : (AUC = 0.788)
6TEST : (AUC = 0.745)
7TEST : (AUC = 0.828)
8TEST : (AUC = 0.689)
9TEST : (AUC = 0.765)
B
Calibration Plot
Observed Proportion
Predicted Probability
BR10TEST (0.137)
BR1TEST (0.136)
BR2TEST (0.138)
BR3TEST (0.14)
BR4TEST (0.141)
BR5TEST (0.141)
BR6TEST (0.132)
BR7TEST (0.137)
BR8TEST (0.131)
BR9TEST (0.139)
E
Calibration Plot
Observed Proportion
Predicted Probability
10TEST (0.15)
1TEST (0.145)
2TEST (0.125)
3TEST (0.116)
4TEST (0.14)
5TEST (0.135)
6TEST (0.153)
7TEST (0.131)
8TEST (0.137)
9TEST (0.109)
C
DCA Curve Plot
Net Benefit
Risk threshold
0.6
0.4
0.2
0.0
-0.2
BR10TEST
BR1TEST
BR2TEST
BR3TEST
BR4TEST
BR5TEST
BR6TEST
BR7TEST
BR8TEST
BR9TEST
Standard
Treat All
Treat None
F
DCA Curve Plot
Net Benefit
Risk threshold
10TEST
1TEST
2TEST
3TEST
4TEST
5TEST
6TEST
7TEST
8TEST
9TEST
Standard
Treat All
Treat None

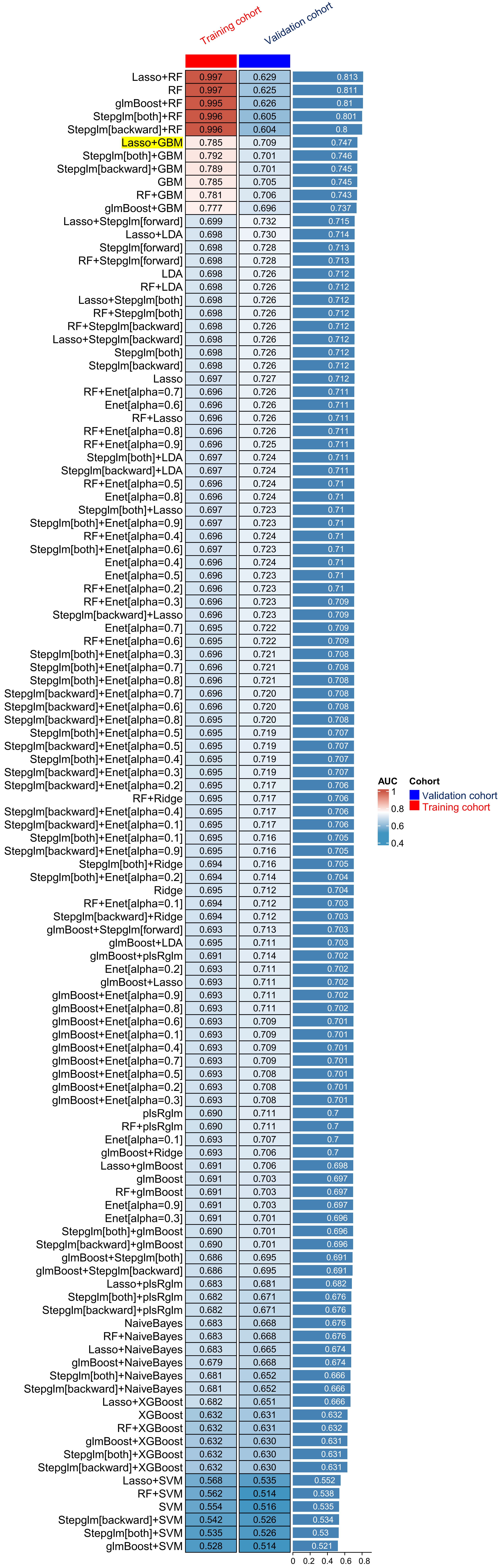
Training cohort
Validation cohort
Lasso+RF 0.997 0.629 0.813
RF 0.997 0.625 0.811
glmBoost+RF 0.995 0.626 0.81
Stepglm[both]+RF 0.996 0.605 0.801
Stepglm[backward]+RF 0.996 0.604 0.8
Lasso+GBM 0.785 0.709 0.747
Stepglm[both]+GBM 0.792 0.701 0.746
Stepglm[backward]+GBM 0.789 0.701 0.745
GBM 0.785 0.705 0.745
RF+GBM 0.781 0.706 0.743
glmBoost+GBM 0.777 0.696 0.737
Lasso+Stepglm[forward] 0.699 0.732 0.715
Lasso+LDA 0.698 0.730 0.714
Stepglm[forward] 0.698 0.728 0.713
RF+Stepglm[forward] 0.698 0.728 0.713
LDA 0.698 0.726 0.712
RF+LDA 0.698 0.726 0.712
Lasso+Stepglm[both] 0.698 0.726 0.712
RF+Stepglm[both] 0.698 0.726 0.712
RF+Stepglm[backward] 0.698 0.726 0.712
Lasso+Stepglm[backward] 0.698 0.726 0.712
Stepglm[both] 0.698 0.726 0.712
Stepglm[backward] 0.698 0.726 0.712
Lasso 0.697 0.727 0.712
RF+Enet[alpha=0.7] 0.696 0.726 0.711
Enet[alpha=0.6] 0.696 0.726 0.711
RF+Lasso 0.696 0.726 0.711
RF+Enet[alpha=0.8] 0.696 0.726 0.711
RF+Enet[alpha=0.9] 0.696 0.725 0.711
Stepglm[both]+LDA 0.697 0.724 0.711
Stepglm[backward]+LDA 0.697 0.724 0.711
RF+Enet[alpha=0.5] 0.696 0.724 0.71
Enet[alpha=0.8] 0.696 0.724 0.71
Stepglm[both]+Lasso 0.697 0.723 0.71
Stepglm[both]+Enet[alpha=0.9] 0.697 0.723 0.71
RF+Enet[alpha=0.4] 0.696 0.724 0.71
Stepglm[both]+Enet[alpha=0.6] 0.697 0.723 0.71
Enet[alpha=0.4] 0.696 0.724 0.71
Enet[alpha=0.5] 0.696 0.723 0.71
RF+Enet[alpha=0.2] 0.696 0.723 0.71
RF+Enet[alpha=0.3] 0.696 0.723 0.709
Stepglm[backward]+Lasso 0.696 0.723 0.709
Enet[alpha=0.7] 0.695 0.722 0.709
RF+Enet[alpha=0.6] 0.695 0.722 0.709
Stepglm[both]+Enet[alpha=0.3] 0.696 0.721 0.708
Stepglm[both]+Enet[alpha=0.7] 0.696 0.721 0.708
Stepglm[both]+Enet[alpha=0.8] 0.696 0.721 0.708
Stepglm[backward]+Enet[alpha=0.7] 0.696 0.720 0.708
Stepglm[backward]+Enet[alpha=0.6] 0.696 0.720 0.708
Stepglm[backward]+Enet[alpha=0.8] 0.695 0.720 0.708
Stepglm[both]+Enet[alpha=0.5] 0.695 0.719 0.707
Stepglm[backward]+Enet[alpha=0.5] 0.695 0.719 0.707
Stepglm[both]+Enet[alpha=0.4] 0.695 0.719 0.707
Stepglm[backward]+Enet[alpha=0.3] 0.695 0.719 0.707
Stepglm[backward]+Enet[alpha=0.2] 0.695 0.717 0.706
RF+Ridge 0.695 0.717 0.706
Stepglm[backward]+Enet[alpha=0.4] 0.695 0.717 0.706
Stepglm[backward]+Enet[alpha=0.1] 0.695 0.717 0.706
Stepglm[both]+Enet[alpha=0.1] 0.695 0.716 0.705
Stepglm[backward]+Enet[alpha=0.9] 0.695 0.716 0.705
Stepglm[both]+Ridge 0.694 0.716 0.705
Stepglm[both]+Enet[alpha=0.2] 0.694 0.714 0.704
Ridge 0.695 0.712 0.704
RF+Enet[alpha=0.1] 0.694 0.712 0.703
Stepglm[backward]+Ridge 0.694 0.712 0.703
glmBoost+Stepglm[forward] 0.693 0.713 0.703
glmBoost+LDA 0.695 0.711 0.703
glmBoost+plsRglm 0.691 0.714 0.702
Enet[alpha=0.2] 0.693 0.711 0.702
glmBoost+Lasso 0.693 0.711 0.702
glmBoost+Enet[alpha=0.9] 0.693 0.711 0.702
glmBoost+Enet[alpha=0.8] 0.693 0.711 0.702
glmBoost+Enet[alpha=0.6] 0.693 0.709 0.701
glmBoost+Enet[alpha=0.1] 0.693 0.709 0.701
glmBoost+Enet[alpha=0.4] 0.693 0.709 0.701
glmBoost+Enet[alpha=0.7] 0.693 0.709 0.701
glmBoost+Enet[alpha=0.5] 0.693 0.708 0.701
glmBoost+Enet[alpha=0.2] 0.693 0.708 0.701
glmBoost+Enet[alpha=0.3] 0.693 0.708 0.701
plsRglm 0.690 0.711 0.7
RF+plsRglm 0.690 0.711 0.7
Enet[alpha=0.1] 0.693 0.707 0.7
glmBoost+Ridge 0.693 0.706 0.7
Lasso+glmBoost 0.691 0.706 0.698
glmBoost 0.691 0.703 0.697
RF+glmBoost 0.691 0.703 0.697
Enet[alpha=0.9] 0.691 0.703 0.697
Enet[alpha=0.3] 0.691 0.701 0.696
Stepglm[both]+glmBoost 0.690 0.701 0.696
Stepglm[backward]+glmBoost 0.690 0.701 0.696
glmBoost+Stepglm[both] 0.686 0.695 0.691
glmBoost+Stepglm[backward] 0.686 0.695 0.691
Lasso+plsRglm 0.683 0.681 0.682
Stepglm[both]+plsRglm 0.682 0.671 0.676
Stepglm[backward]+plsRglm 0.682 0.671 0.676
NaiveBayes 0.683 0.668 0.676
RF+NaiveBayes 0.683 0.668 0.676
Lasso+NaiveBayes 0.683 0.665 0.674
glmBoost+NaiveBayes 0.679 0.668 0.674
Stepglm[both]+NaiveBayes 0.681 0.652 0.666
Stepglm[backward]+NaiveBayes 0.681 0.652 0.666
Lasso+XGBoost 0.682 0.651 0.666
XGBoost 0.632 0.631 0.632
RF+XGBoost 0.632 0.631 0.632
glmBoost+XGBoost 0.632 0.630 0.631
Stepglm[both]+XGBoost 0.632 0.630 0.631
Stepglm[backward]+XGBoost 0.632 0.630 0.631
Lasso+SVM 0.568 0.535 0.552
RF+SVM 0.562 0.514 0.538
SVM 0.554 0.516 0.535
Stepglm[backward]+SVM 0.542 0.526 0.534
Stepglm[both]+SVM 0.535 0.526 0.53
glmBoost+SVM 0.528 0.514 0.521
0 0.2 0.4 0.6 0.8
AUC
1
0.8
0.6
0.4
Cohort
Validation cohort
Training cohort
