# Supplementary material for: Development and validation of an explainable machine learning model to predict Delphian lymph node metastasis in papillary thyroid cancer: a large cohort study
Source: J Cancer. 2025 Mar 3;16(6):2041–61. doi: 10.7150/jca.110141 (PMC11905415; doi:10.7150/jca.110141)
Supplement: Supplementary file 1 — Supplementary figures and tables. [file jcav16p2041s1.zip › Supplementary appendix.docx]

**Supplementary methods.**

**Surgery**

Surgical interventions followed established Chinese thyroid cancer guidelines [14,15]. Complete thyroid removal was implemented for bilateral PTC cases. For single-sided PTC instances, physicians performed either complete thyroid extraction or partial removal with isthmus excision. In unilateral situations meeting specific criteria (tumour diameter exceeding 4 cm, multiple foci within one lobe, extension beyond thyroid boundaries, or remote spread)—confirmed via rapid tissue analysis and pre-surgical screening—complete thyroid removal with isthmus excision was considered, adhering to Chinese Thyroid Association standards. Standard procedure included dissection of pretracheal, paratracheal (same-side or bilateral), and surrounding adipose tissues. Surgeons extracted identified DLNs during procedures. LN-negative PTC cases underwent same-side prophylactic CND (pCND). Detection of suspicious cervical nodes through pre-operative ultrasound, physical examination, or intra-operative observation prompted contralateral CND execution.

In order to improve the detection rate of DLNs, we performed the following operations: 1. Nanocarbon labelling of negatively developed lymph nodes, intraoperative exposure of thyroid gland lobes and isthmus tissues, injection of a small amount of nanocarbon suspension at multiple points in the lobes and isthmus of the thyroid gland, waiting for about 5 minutes, and then sweeping the DLNs after LNs are developed. 2. In terms of the surgical approach, prior to the sweeping of the DLNs, the thyroid pulling hook is used to pull both sides of the thyroid to expose the anterior larynx completely, then the DLNs are cleaned thoroughly, and the DLN specimens are sent individually for pathological examination before thyroidectomy and clearing of the pretracheal and paratracheal LNs are performed.

**Data extraction and outcomes**

For each participant, preoperative baseline characteristics were procured through the hospital’s HIS medical platform. The collected information encompassed age; gender (male and female); time to diagnose the tumor; thyroid stimulating hormone (TSH); thyroglobulin (TG); anti-thyroglobulin antibodies (TGAB); triiodothyronine (T3); thyroxine (T4); free triiodothyronine (FT3); free thyroxine (FT4); B ultrasound report: tumor size (the maximum diameter for a single node; the maximum diameters sum for multiple nodes), tumor site (upper, middle, below, isthmus, and multiple sites), bilateral tumor (no and yes), close to anterior capsule (no and yes), close to posterior capsule (no and yes), close to trachea (no and yes), left paratracheal LN (CLN) suspicious metastasis (no and yes), left 2 region LN suspicious metastasis (no and yes), left 3 region LN suspicious metastasis (no and yes), left 4 region LN suspicious metastasis (no and yes), right CLN suspicious metastasis (no and yes), right 2 region LN suspicious metastasis (no and yes), right 3 region LN suspicious metastasis (no and yes), right 4 region LN suspicious metastasis (no and yes); computed tomography (CT) report: CLN suspicious metastasis (no and yes), lateral cervical LN suspicious metastasis (no and yes), superior mediastinum LN suspicious metastasis (no and yes), lung suspicious metastasis (no and yes). The B ultrasound and CT results are issued by two imaging physicians, one of whom has more than 10 years of experience.

Additionally, detailed postoperative pathologic features were extracted, including tumour size, PTC subtype (follicular and classical), fibrosis (no and yes), small foci of squamous lesions (no and yes), hashimoto thyroiditis (no and yes), pathological tumour size (pT) stage (T1a, T1b, T2, T3a, T3b, and T4a), pathological node (pN) stage (N0, N1a, and N1b), pathological metastasis (pM) stage (M0 and M1), pathological tumour node metastasis (pTNM) stage (I, II, III, and IV), multifocal (no and yes), vascular invasion (no and yes), intra-glandular dissemination (no and yes), capsular invasion (no and yes), extracapsular spread (no and yes), trachea invasion (no and yes), nerve invasion (no and yes), superior mediastinal metastasis (no and yes). The pathological reports are issued by two imaging physicians, one of whom has more than 10 years of experience. Following the American Joint Committee on Cancer (AJCC) tumour-node-metastasis (TNM) staging system eighth edition [16], PTC TNM staging was ascertained primarily through pathological evidence and secondarily through imaging findings.
